# Broomeanamides: Cyclic Octapeptides from an Isolate of the Fungicolous Ascomycete *Sphaerostilbella broomeana* from India

**DOI:** 10.1021/acs.jnatprod.1c00414

**Published:** 2021-06-30

**Authors:** Dulamini I. Ekanayake, Bruno Perlatti, Dale C. Swenson, Kadri Põldmaa, Gerald F. Bills, James B. Gloer

**Affiliations:** † Department of Chemistry, University of Iowa, Iowa City, Iowa 52242, United States; ‡ Texas Therapeutic Institute, The Brown Foundation Institute of Molecular Medicine, University of Texas Health Science Center, 1881 East Road, Houston, Texas 77054, United States; § Department of Botany, Institute of Ecology and Earth Sciences, University of Tartu, Lai 40, EE-51005 Tartu, Estonia

## Abstract

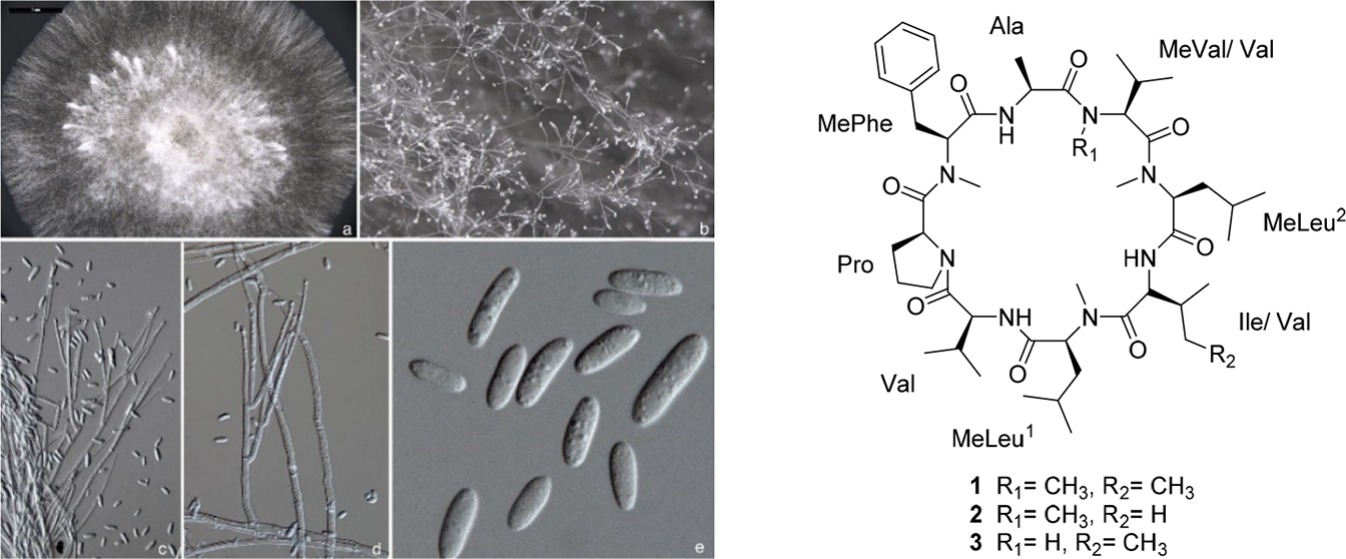

The genus *Sphaerostilbella* comprises fungi that colonize basidiomata of wood-inhabiting fungi, including important forest pathogens. Studies of fermentation cultures of an isolate (TFC201724) collected on the foothills of Himalayas, and closely related to *S. broomeana* isolates from Europe, led to the identification of a new cyclic octapeptide along with two closely related analogues (**1**–**3**) and four dioxopiperazines (**4**–**7**). The structure of the lead compound, broomeanamide A (**1**), was assigned mainly by analysis of 2D NMR and HRESIMS data. The structure consisted of one unit each of *N*-MeVal, Ala, *N*-MePhe, Pro, Val, and Ile and two *N*-MeLeu units. The amino acid sequence was determined on the basis of 2D NMR and HRESIMSMS data. NMR and HRMS data revealed that the other two new peptides have the same amino acid composition except that the Ile unit was replaced with Val in one instance (**2**) and the *N*-MeVal unit was replaced with Val in the other (**3**). The absolute configuration of **1** was assigned by analysis of the acid hydrolysate by application of Marfey’s method using both C_18_ and C_3_ bonded-phase columns. Broomeanamide A (**1**) showed antifungal activity against *Cryptococcus neoformans* and *Candida albicans*, with MIC values of 8.0 and 64 μg/mL, respectively.

The high rate of morbidity and mortality associated with microbial infections and the development of multidrug resistance by infectious agents continue to intensify the need for new antimicrobial agents. The proven track record of fungi makes them an attractive resource for the discovery of novel compounds with antimicrobial activity. Fungicolous fungi, which colonize other fungi, are well known for their ability to produce antimicrobial secondary metabolites.^1,2^

Among ascomycetes, the family Hypocreaceae (Hypocreales) includes the highest diversity of fungicolous fungi, the majority of which occur on wood-decaying basidiomycetes. The genus *Sphaerostilbella* is exceptional in the family because of the tendency to specialize on wood-inhabiting members of the Russulales, an order well known for its agaric members from the large genera of *Russula* and *Lactarius*. One of these species, *S. broomeana*, grows exclusively on basidiomata of *Heterobasidion* species, some of which are major forest pathogens. It was known only from Europe until a morphologically very similar, but genetically distinct anamorph was recently collected on the foothills of the Himalayas in India.^3^ Fermentation of this *Sphaerostilbella* isolate (TFC201724) afforded an antifungal extract, which was found to contain three new cyclic octapeptides (**1**–**3**) along with four dioxopiperazines (**4**–**7**). Among the latter, **4** and **6** do not appear to have been previously encountered from a natural source, although **4** has been reported as a synthetic product.^4^ The most abundant cyclic octapeptide metabolite (**1**) showed significant antifungal activity.

Relatively few cyclic octapeptides have been reported from fungi, but such compounds show a wide range of bioactivities. For examples, the cyclic octapeptide fungisporin, produced by various *Penicillium* and *Aspergillus* spp., is considered to be a mycotoxin.^5^ Epichlicin, isolated from the endophytic fungus *Epichloë typhina*, exhibited potent inhibitory activity toward spore germination of *Cladosporium phlei*, a fungal pathogen of the timothy plant, with an IC_50_ value of 22 nM.^6^ Shearamide A was isolated from the stromata of *Eupenicillium shearii* (now *Talaromyces pinophilus*) and displayed insecticidal effects against *Helicoverpa zea* larvae.^7^ Two other new cyclic octapeptides, mariannamides A and B, have been reported from *Mariannaea elegans* and showed antimicrobial activity against *Escherichia coli* and *Cryptococcus neoformans*.^8^

Recently, we reported a pair of peptaibol-type metabolites called sphaerostilbellins A and B from an isolate of *S. toxica*,^9^ a species closely related to the isolate used in this study.^3^ To our knowledge, there are no other reports of secondary metabolites from any member of *Sphaerostilbella*. Herein, we describe the structure elucidation and characterization of **1**–**7**, as well as the results of antibacterial and antifungal assays for a subset of these compounds.

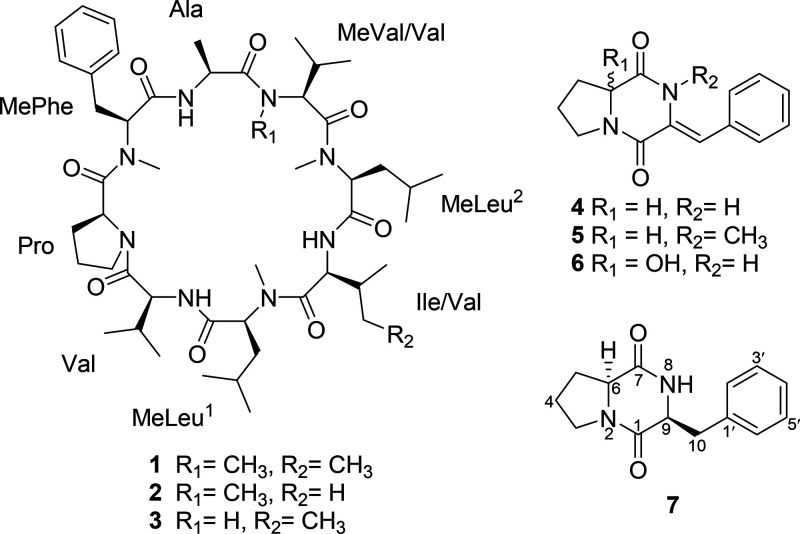



## Results and Discussion

A solid-substrate rice fermentation of *S. broomeana* TFC201724 was extracted with EtOAc, and the resulting extract was partitioned between acetonitrile and hexanes. The acetonitrile-soluble fraction was subjected to silica gel column chromatography and reversed-phase HPLC to afford broomeanamides A (**1**), B (**2**), and C (**3**) and dioxopiperazines **4**–**7**.

**Figure 1:**
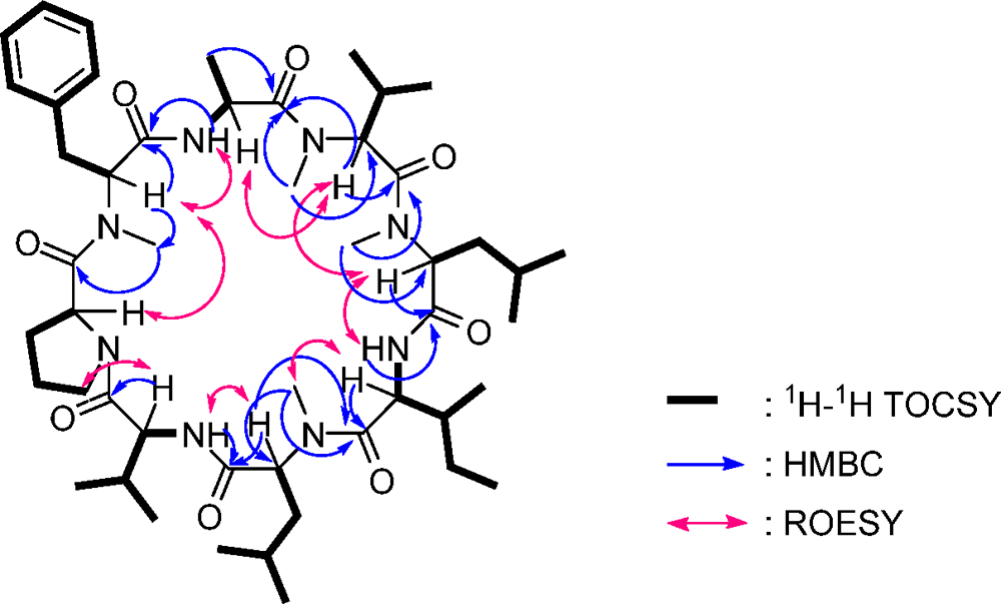
Selected 2D NMR correlations for broomeanamide A (**1**).

Broomeanamide A (**1**) was obtained as a white powder with a molecular formula of C_49_H_80_N_8_O_8_ (14 degrees of unsaturation), as determined by HRESIMS. Characteristic signals observed in the ^1^H NMR spectrum (CDCl_3_; Table 1) indicated that **1** is a peptide. Analysis of its ^1^H and ^13^C NMR and HSQC data revealed the presence of three amide *N*-H protons, 15 methyl groups (including four *N*-methyls), six methylenes, 14 methines (eight of which are heteroatom-bonded), one phenyl group, and eight carboxylic carbons (δ_C_ 168.3–174.8). Interpretation of ^1^H–^1^H TOCSY (Figure 1) and HSQC data of **1** established the presence of individual valine (Val), alanine (Ala), proline (Pro), isoleucine (Ile), *N*-methylvaline (*N*-MeVal), and *N*-methylphenylalanine (*N*-MePhe) units, along with two *N*-methylleucine (*N*-MeLeu^1^, *N*-MeLeu^2^) residues. These data accounted for all the NMR resonances of **1** and 13 of the 14 unsaturations, indicating that **1** is a cyclic octapeptide.

**Table 1:**
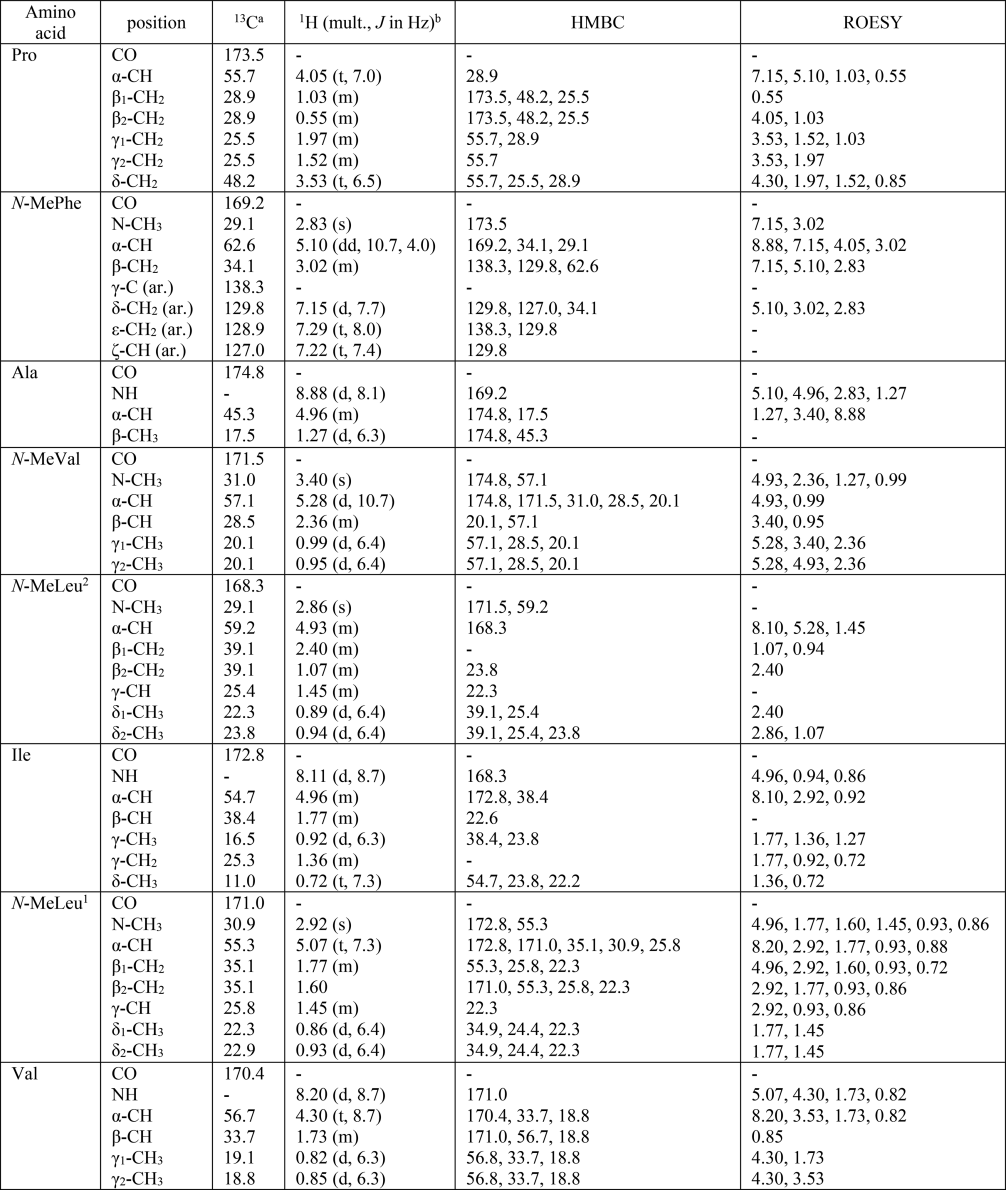
NMR Spectroscopic Data for Broomeanamide A (**1**) in CDCl__3__

a 150 MHz; CDCl_3_ signal reference set at δ 77.2.

b 600 MHz; residual CHCl_3_ signal reference set at δ 7.26.

The complete amino acid sequence was determined by extensive analysis of HMBC and ROESY correlations (Figure 1), supported by HRESIMSMS data (Figure S10). Interpretation of the NMR data collected in CDCl_3_ was complicated somewhat by overlap of three α-proton signals at δ_H_ 4.96, which were identified as those of Ala, Ile, and *N*-MeLeu^2^ on the basis of the TOCSY data, but other resonances were generally well-resolved, and collection of the full 2D NMR data set overcame this issue. The carbonyl resonance of Ala (δ_C_ 174.8) and its α-carbon (δ_C_ 45.3) were identified based on HMBC correlations of the β-CH_3_ of Ala to δ_C_ 174.8 and δ_C_ 45.3. The *N*-MePhe unit was acylated by Pro based on an HMBC correlation from the N-CH_3_ signal of *N*-MePhe to the Pro carbonyl, which was assigned by HMBC correlations with the Pro β-proton signals. The latter were somewhat upfield shifted relative to typical Pro signals, possibly due to shielding associated with spatial proximity to the aryl group. This connection was supported by ROESY correlations between the α-protons of the *N*-MePhe and Pro units. An HMBC correlation from the Ala amide NH to the carbonyl carbon of the *N*-MePhe unit and a ROESY correlation between the α-proton of *N*-MePhe and the amide NH of Ala indicated that *N*-MePhe acylated Ala in the sequence. HMBC correlations of the α-proton and the N-CH_3_ of *N*-MeVal to the carbonyl of Ala extended the sequence by indicating acylation of the *N*-MeVal unit by the Ala unit. A ROESY correlation from the overlapped signal for the Ala unit α-proton to the α-proton of the *N*-MeVal was consistent with this connection, completing the partial sequence Pro → *N*-MePhe → Ala → *N*-MeVal. This was further supported by observation of an *m*/*z* 443.2647 ion in the HRESIMSMS data corresponding to the expected C_24_H_35_N_4_O_4_ fragment. The *N*-MeLeu^2^ unit was acylated by *N*-MeVal based on an HMBC correlation from the *N*-MeLeu^2^ N-CH_3_ signal to the carbonyl of *N*-MeVal. HMBC correlations from the Ile NH to carbonyls at δ 168.3 and 172.8 and of the N-CH_3_ signal of *N*-MeLeu^1^ to the carbonyl at δ 172.8 indicated that Ile acylates the *N*-MeLeu^1^ unit and identified the Ile carbonyl shift as δ 168.3. HMBC correlations from the α-proton of *N*-MeLeu^2^ to the carbonyl of Ile further extended the partial sequence to Pro → *N*-MePhe → Ala → *N*-MeVal → *N*-MeLeu^2^ → Ile → *N*-MeLeu^1^. This extended connectivity was supported by HRESIMSMS ions at *m*/*z* 683.4479 and 810.5466, consistent with the formulas for the sequence through Ile and through *N*-MeLeu^1^, respectively. Additional ROESY correlations were consistent with this connectivity. This effectively completed the structure, as the only remaining unit to insert was the Val residue, and its location between the *N*-MeLeu^1^ and Pro units was supported by an HMBC correlation from the Val amide NH to the carbonyl carbon of the *N*-MeLeu^1^ unit and a ROESY correlation between the α-proton of *N*-MeLeu^1^ and the amide NH of Val, as well as a ROESY correlation between the α-proton of Val and the δ-protons of Pro, indicating that Val acylated Pro in the sequence. Thus, the gross structure of broomeanamide A was assigned as shown in structure **1**.

Marfey’s method^9,10^ was applied to assign the absolute configurations of the amino acid residues resulting from acid hydrolysis of broomeanamide A. The 1-fluoro-2,4-dinitrophenyl-5-l-alanine amide derivatives of the amino acids present in the acid hydrolysate of **1** were analyzed by LC-MS along with those of authentic d- and l-amino acids. Initial efforts employed a C_18_ column and did not resolve all of the derivatives, a problem that is known to arise, especially for certain *N*-methyl amino acids. However, complementary chromatography on a C_3_ column^11^ led to separation of those that were unresolved on C_18_, ultimately enabling assignment of the l-configuration for all of the amino acid residues (Figure S11).

Broomeanamides B (**2**) and C (**3**) were obtained in considerably lesser amounts. The molecular formula of broomeanamide B (**2**) was determined to be C_48_H_78_N_8_O_8_ (14 degrees of unsaturation) by HRESIMS, indicating the presence of one CH_2_ unit less than that of **1**.

Analysis of the ^1^H NMR spectroscopic data (Table S1) confirmed that **2** was a close analogue of **1** with the same amino acid composition except that the Ile unit was replaced with Val. Specifically, the data showed the appearance of two methyl doublets at δ_H_ 0.76 (δ_C_ 17.9) and δ_H_ 0.88 (δ_C_ 14.1) in place of the methyl triplet at δ_H_ 0.72 (δ_C_ 11.0) and the methyl doublet at δ_H_ 0.72 (δ_C_ 16.5) for the Ile residue in **1**. The α-proton signals were somewhat better resolved in this case, but the sequence was otherwise identical, as supported by nearly identical NMR shifts for the remainder of the molecule. The TOCSY, HSQC, and HMBC results were also consistent with the structure proposed for **2**.

The small sample of broomeanamide C (**3**) obtained was not completely freed of minor homologues, but could be identified based on NMR and MS data, as it was clearly closely related to **1** and **2**. HRESIMS data indicated that it is an isomer of **2**. The ^1^H NMR spectrum of **3** (Table S2) was similar to that of **1** except for the appearance of a new amide *N*-H doublet at δ_H_ 6.65 and the absence of the *N*-methyl signal corresponding to the *N*-MeVal unit in **1**, revealing that the *N*-MeVal residue in **1** was replaced by a Val unit in **3**.

Several dioxopiperazines were also isolated from these cultures. Dioxopiperazines are frequently encountered from fungi, bacteria, and plants and display a variety of biological activities. Analysis of NMR and MS data and comparison with literature values enabled identification of the gross structures of dioxopiperazines **4**, **5**, and **6**, all of which contain a dehydrophenylalanine unit. Among these, only **5** had been previously reported from a natural source, although compound **4** had been described as a synthetic product.^4^ The olefin geometry in such compounds is sometimes not discussed in literature reports, although it has been shown that the chemical shift of the olefinic signal is somewhat diagnostic for such assignments, with the olefinic ^1^H NMR signal for the *Z*-isomer typically appearing ca. 0.5 ppm downfield relative to that of the *E*-isomer in cases where both isomers are available for comparison.^12^ The double bond in **4** between C-9 and C-10 was confirmed to have the *Z*-geometry on the basis of a NOESY correlation between NH-8 and the H-2′/6′ resonance of the aromatic ring, as well as the olefinic ^1^H NMR shift (δ_H_ 6.99).

The absolute configuration at C-6 in **4** was not initially assigned, as there was no [α]_D_ literature value for direct comparison. Crystals of **4** were later obtained, enabling analysis by X-ray crystallography with an eye toward unambiguously assigning the configuration. Interestingly, in addition to confirming the gross structure and the olefin geometry, these data revealed that the sample of **4** was obtained as a racemate. Notably, closely related compound **5** was also obtained from this extract and identified as the N-8 methyl analogue of **4**, which had been previously reported from *Penicillium pinophilum* and assigned the 6*S* configuration.^13^ Prior to that, **5** had also been reported as a synthetic product.^14^^1^H NMR and MS data matched well with literature values for **5**, and the [α]_D_ had the same sign as that reported for the 6*S* isomer, though it was significantly lower in magnitude.^13,14^ Upon analysis of these literature descriptions, however, some confusing issues were noted. First of all, both prior references show structures clearly depicting the 6*R* configuration even though the text indicates that the assignment was 6*S* in each case. Presumably, this was a graphical typo that was carried over in the second report, which referenced the first. Moreover, the earlier report described synthetic **5**, for which a [α]_D_ value of +535 was reported using CHCl_3_ as solvent. The later report (of naturally occurring **5** from *P. pinophilum* as noted above) gave a value of +90 using MeOH as solvent. Measurements of our sample gave values of +15 and +20 in CHCl_3_ and MeOH, respectively. The rather large discrepancies among these numbers could be explained by varying levels of epimerization at C-6 in the two nonsynthetic samples. Other literature reports describe some tendency for Pro-containing dioxopiperazines to epimerize under certain conditions.^15^ Given the finding that **4** crystallized as a racemate, it seems that the sample of **5** obtained was likely scalemic. It may be that the sample of **4** initially obtained was also scalemic, but that there is some preference for crystallization in the racemic form.

**Figure 2:**
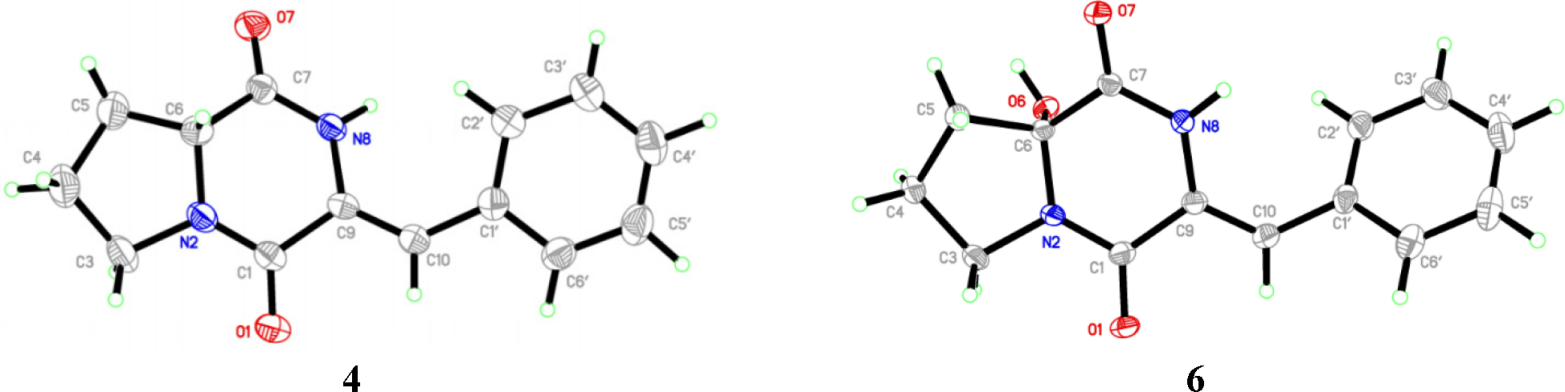
X-ray models of compounds **4** and **6**.

Related compound **6** was also obtained, differing from **4** by hydroxylation at C-6. Although **6** does not appear to have been reported previously, close analogues have been described from a plant source (*Claoxylon polot*) that bear a hydroxy group or methoxy group at C-6 of the proline residue and methyl groups at the C-4 and N-8 positions.^16^ Compound **6** crystallized from MeOH and was also subjected to X-ray crystallographic analysis, leading to confirmation of the structure and to recognition that it was also obtained as a racemate. This was perhaps less unexpected given that the α-position of the Pro unit is modified. ORTEP representations of **4** and **6** are shown in Figure 2. Compound **7** was identified as the well-known cyclo(l-Pro-l-Phe) by comparing the ^1^H NMR, MS, and specific rotation data with literature values.^17^

In standard disk assays against *Candida albicans* (ATCC 10231) and *Staphylococcus aureus* (ATCC 29213), broomeanamides A and B (**1** and **2**) both showed inhibition at the 50 μg/disk level against *S. aureus,* while broomeanamide A displayed inhibition against *C. albicans* at the 50 μg/disk level. Compounds **4**–**7** showed no activity in these screens. Broomeanamide A (**1**) was separately tested for antifungal activity against *Cryptococcus neoformans* (H99), *C. albicans*, and *S. aureus* in MIC assays (Table 2). Broomeanamide A displayed a significant inhibitory effect against *C. neoformans*, with an MIC value of 8 μg/mL at 37 °C, while showing lesser inhibitory effects against *C. albicans* and *S. aureus* with MIC values of 64 and 128 μg/mL, respectively (Figure S20).

**Table 2:** Minimum Inhibitory Concentration Assay Results for **1**

	compound (MIC; μg/mL)	
organism	**1**	control^a^
*Staphylococcus aureus* ATCC 43300	128	0.16
*Candida albicans* ATCC 10231	64	1.70
*Cryptococcus neoformans* H99 (23 °C)	128	0.85
*Cryptococcus neoformans* H99 (37 °C)	8	0.85

a Chlortetracycline + streptomycin was the control for *S. aureus*. Amphotericin B was the control for fungal strains.

## Experimental Section

### General Experimental Procedures

Specific rotations were measured on an AUTOPOL III automatic polarimeter. ^1^H and ^13^C NMR spectra were recorded using Bruker AVANCE-600 or AVANCE-500 spectrometers. Chemical shift values were referenced to residual solvent signals for CDCl_3_ (δ_H_/δ_C_, 7.26/77.2). HSQC, HMBC, TOCSY, and ROESY data were recorded using the Bruker AVANCE-600 instrument. HRESIMS and HRESIMSMS data were obtained using a Waters Q-Tof Premier mass spectrometer. Semipreparative HPLC separations were carried out using an Agilent 1260 Infinity LC system instrument with a diode array detector equipped with a semipreparative Apollo C_18_ column (Grace, 1.0 × 25 cm, 5 μm) with UV detection at 210, 254, and 350 nm or an Agilent 1220 Infinity LC system instrument equipped with the same column type under UV detection at 254 nm. Conditions for analytical separation of Marfey derivatives are described below. Single-crystal X-ray data were collected using a Bruker D8-Venture Duo diffractometer equipped with a Photon III detector, Mo-target and Cu-target microfocus X-ray tubes, and an Oxford Cryostreams 800 series N_2_ gas stream sample cooler/heater.

### Fungal Material and Fermentation

The culture of *S. broomeana* TFC201724 was isolated from mycelium effused on an old basidioma of *Heterobasidion* cf. *linzhiense* (*H. insulare* group) growing on a decaying stump. The material was collected by one of the authors (K.P.) in a cedar forest 4 km west of Dhanaulti, Dehradun district, Uttarakhand, India, on October 9, 2012, and deposited at the fungarium of the University of Tartu (TUF119036). It included only an anamorph with abundant conidia that were isolated, and colonies were grown on 1.5% malt extract agar (MEA, Oxoid, Cambridge, UK) in the dark at 24 °C. The culture has been deposited at the Tartu Fungal Culture Collection (TFC) of the University of Tartu, and the TEF (MH795104) and ITS-LSU rDNA (MH795096) sequences are available in GenBank.^3^ Maximum likelihood analyses of the combined rDNA and TEF sequences of the *S. broomeana* group^3^ in RAxML,^18^ applying the default settings, revealed TFC201724 to form the sister group of isolates of *S. broomeana* originating from Europe (Figure S2). A subculture on PDA was used to inoculate 10 1-L Erlenmeyer flasks, each containing 100 g of autoclaved rice in 100 mL of distilled water.^19^ Two 0.25 cm^2^ agar plugs from the subculture were aseptically transferred into each flask, and the cultures were incubated statically at room temperature for 30 days.

### Extraction and Isolation

Each fermented flask was extracted with EtOAc (0.2 L × 2) and filtered, and the combined filtrate was air-dried to yield a crude extract (1.5 g). The extract was partitioned between hexanes (10 mL) and MeCN (10 mL) to obtain hexanes and MeCN fractions. Upon evaporation of solvent, these samples were found to contain 0.4 and 1.1 g, respectively. A small portion of the MeCN fraction (14.4 mg) was subjected directly to semipreparative RP-HPLC (Agilent 1220 Infinity-C_18_ column; 5 μm; 9.4 × 250 mm; gradient elution 40–100% MeCN in H_2_O over 50 min; 2 mL/min) to afford **7** (2.0 mg, *t*_R_ 9.2 min), **4** (1.1 mg, *t*_R_ 11.3 min), and **5** (0.8 mg, *t*_R_ 17.4 min), indicating that they were major components of the extract.

The ^1^H NMR spectrum of the HPLC fraction 14 (0.6 mg, *t*_R_ 42.2 min) showed complex signals characteristic of a mixture. Therefore, a larger portion of the MeCN fraction (339 mg) was subjected to silica gel CC using gradient elution with hexanes/EtOAc–MeOH to yield 11 fractions. Fraction 4 (45.7 mg), eluted with 75% EtOAc in hexanes, was further purified by semipreparative RP-HPLC as above to afford **1** (5.0 mg, *t*_R_ 42.2 min) and **2** (0.5 mg, *t*_R_ 45.4 min). Fraction 3 (104 mg), eluted with 50% EtOAc in hexanes, was further separated by semipreparative RP-HPLC (Agilent 1260 Infinity-C_18_ column; 5 μm; 9.4 × 250 mm; gradient elution 40–100% MeCN in H_2_O over 60 min; 2 mL/min) to afford **6** (1.5 mg, *t*_R_ 9.1 min), and fraction 5 (43 mg), eluted with 100% EtOAc in hexanes, was further separated by semipreparative RP-HPLC (Agilent 1260 Infinity-C_18_ column; 5 μm; 9.4 × 250 mm; gradient elution 40–100% MeCN in H_2_O over 60 min; 2 mL/min) to afford **3** (0.5 mg, *t*_R_ 39.0 min). Known compounds **5** and **7** were identified by comparison of their NMR and specific rotation data with literature values.^13,17^

#### Broomeanamide A (**1**):

white solid; [α]^20^_D_ −6 (*c* 0.05, MeOH); ^1^H and ^13^C NMR and HMBC data, see Table 1; positive ion HRESIMS *m*/*z* 909.6197 [M + H]^+^ (calcd for C_49_H_80_N_8_O_8_ + H, 909.6177).

#### Broomeanamide B (**2**):

white solid; [α]^20^_D_ −18 (*c* 0.03, MeOH); ^1^H and ^13^C NMR and HMBC data, see Table S1; positive ion HRESIMS *m*/*z* 895.6032 [M + H]^+^ (calcd for C_48_H_78_N_8_O_8_ + H, 895.6021).

#### Broomeanamide C (**3**):

white solid; [α]^20^_D_ −42 (*c* 0.03, MeOH); ^1^H and ^13^C NMR and HMBC data, see Table S2; negative ion HRESIMS *m*/*z* 893.5887 [M – H]^−^ (calcd for C_48_H_78_N_8_O_8_ – H, 893.5864).

#### Compound **4**:

white solid; ^1^H NMR (CDCl_3_, 500 MHz) δ 7.74 (1H, br s, NH-8), 7.43 (2H, br t, *J* = 7.3 Hz, H-3′, 5′), δ 7.37 (2H, br d, *J* = 7.6 Hz, H-2′, 6′), 7.34 (1H, br t, *J* = 7.3 Hz, H-4′), 6.99 (1H, s, H-10), 4.31 (1H, dd, *J* = 10.2, 6.5 Hz, H-6), 3.82 (1H, m, H-3a), 3.65 (1H, m, H-3b), 2.47 (1H, m, H-5a), 2.10 (1H, m, H-5b), 2.03 (1H, m, H-4a), 1.96 (1H, m, H-4b); ^13^C NMR (CDCl_3_, 150 MHz) 166.0 (C, C-7), 158.0 (C, C-1), 133.3 (C, C-9), 129.5 (CH, C-2′, 6′), 128.8 (C, C-1′), 128.6 (CH, C-3′, 5′), 127.5 (CH, C-4′), 116.0 (CH, C-10), 59.3 (CH, C-6), 45.8 (CH_2_, C-3), 29.1 (CH_2_, C-5), 22.0 (CH_2_, C-4), negative ion HRESIMS *m*/*z* 241.0977 [M – H]^−^) (calcd for C_14_H_14_N_2_O_2_ – H, 241.0978).

#### Compound **6**:

white solid; ^1^H NMR (CDCl_3_, 500 MHz) δ 7.74 (1H, br s, NH-8), 7.43 (2H, br t, *J* = 7.6 Hz, H-3′, 5′), δ 7.39 (2H, br d, *J* = 7.3 Hz, H-2′, 6′), 7.35 (1H, br t, *J* = 7.3 Hz, H-4′), 7.06 (1H, s, H-10), 3.89 (1H, m, H-3a), 3.77 (1H, m, H-3b), 3.19 (1H, s, OH-6), 2.38 (1H, m, H-5a), 2.29–2.21 (2H, m, H-5b, H-4a), 2.08 (1H, m, H-4b); ^13^C NMR (CDCl_3_, 150 MHz) 165.1 (C, C-7), 158.3 (C, C-1), 133.0 (C, C-9), 129.6 (CH, C-2′, 6′), 129.1 (C, C-1′), 128.7 (CH, C-3′, 5′), 126.6 (CH, C-4′), 117.7 (CH, C-10), 87.2 (C–OH, C-6), 45.8 (CH_2_, C-3), 36.8 (CH_2_, C-5), 19.5 (CH_2_, C-4), negative ion HRESIMS *m*/*z* 257.0921 [M – H]^−^ (calcd for C_14_H_14_N_2_O_3_ – H, 257.0926).

### Marfey’s Analysis of Broomeanamide A

Determination of the absolute configuration of the amino acid units in **1** was accomplished using Marfey’s method in conjunction with both C_3_ and C_18_ chromatographic separation.^9−11^ For the derivatization reaction, 0.2 mg of **1** was transferred to a 2 mL glass tube, to which 500 μL of 6 M HCl was added and kept at 110 °C for 16 h. After hydrolysis, the liquid was evaporated under a stream of air, and 50 μL of H_2_O, 20 μL of 1 M NaHCO_3_, and 100 μL of 1% Marfey’s reagent (1-fluoro-2,4-dinitrophenyl-5-l-alanine amide, l-FDAA) in acetone were added. The tube was sealed and kept at 40 °C with occasional agitation. The reaction was quenched with addition of 40 μL of 2 M HCl and dried under a stream of air. The reaction product was dissolved in 200 μL of MeOH, filtered with a 0.22 μm hydrophilic PTFE filter, and submitted to C_3_ and C_18_ analysis using an Agilent 1260 HPLC coupled to an Agilent 6120 single quadrupole MS, collecting positive and negative ESIMS at *m*/*z* 160–1500. Mobile phases consisted of 0.1% formic acid in MeCN (A), 0.1% aqueous formic acid (B), and 0.1% formic acid in MeOH (C). C_3_ chromatography employed a Zorbax SB-C3 column (150 × 4.6 cm, 5 μm), with a 55 min gradient elution from 5:80:15 to 5:35:60 A:B:C, with a column temperature of 50 °C. For separation of l-FDAA-N-MePhe isomers, an Ace Equivalence C_18_ column (150 × 4.6 mm, 5 μm) maintained at 40 °C was employed, using a gradient of 20:80 to 50:50 A:B over 45 min. Authentic standards of both d- and l-isomers of alanine (Ala), valine (Val), proline (Pro), isoleucine (Ile), *allo*-isoleucine (allo-Ile), *N*-methyl-valine (N-MeVal), *N*-methyl-leucine (N-MeLeu), and *N*-methyl-phenylalanine (N-MePhe) were subjected to Marfey’s reaction conditions and analyzed using the same LC-MS protocol.

The l-FDAA-l-N-MeVal and l-FDAA-l-*allo*-Ile isomers coeluted and showed the same major *m*/*z* fragment. However, all l-FDAA-Ile derivatives produce another fragment at *m*/*z* 338, which was used to confirm the absence of l-*allo*-Ile. As such, the peak at 33.6 min with *m*/*z* 384 could be characterized as l-FDAA-l-N-MeVal. Thus, all seven amino acids (Ala, Val, Pro, Ile, N-MeLeu, N-MeVal, N-MeLeu, and N-MePhe), each having the l-configuration, were detected in the hydrolysate of **1**.

### X-ray Crystallographic Analysis of **4**

Upon crystallization from CH_3_OH using the vapor diffusion method, colorless crystals were obtained. A crystal (0.020 mm × 0.030 mm × 0.365 mm) was separated from the sample and mounted on a glass fiber, and data were collected using Incoatec microsource 3.0 Cu Kα radiation, λ = 1.541 78 Å at 150(2) K. Crystal data: C_14_H_14_N_2_O_2_, M = 242.27 g/mol, space group tetragonal, *P*42*bc*; unit cell dimensions *a* = 14.8338(3) Å, *b* = 14.8338(3) Å, *c* = 11.0209(4) Å, *V* = 2425.06(13) Å^3^, *Z* = 8, *D*_calcd_ = 1.327 g/cm^3^, μ = 0.733 mm^–1^, *F*(000) = 1024. A total of 3036 frames were collected. The total exposure time was 42.6 h. The frames were integrated with the Bruker SAINT software package using a narrow-frame algorithm. The integration of the data using a tetragonal unit cell yielded a total of 30 899 reflections to a maximum θ angle of 66.62° (0.84 Å resolution), of which 2098 were independent (average redundancy 14.728, completeness = 100.0%, *R*_int_ = 8.86%, *R*_sig_ = 3.22%) and 1920 (91.52%) were greater than 2σ(*F*^2^). Data were corrected for absorption effects using the multi-scan method (SADABS). The ratio of minimum to maximum apparent transmission was 0.856. The calculated minimum and maximum transmission coefficients (based on crystal size) are 0.7760 and 0.9850. The structure was solved and refined using the Bruker SHELXTL software package. The final anisotropic full-matrix least-squares refinement on *F*^2^ with 219 variables converged at *R*_1_ = 4.13% for the observed data and *wR*_2_ = 10.73% % [*I* > 2σ(*I*)] for all data. Crystallographic data for **4** have been deposited with the Cambridge Crystallographic Data Centre (CCDC deposition number 2060793).^20^

### X-ray Crystallographic Analysis of **6**

A colorless plate-like crystal (0.030 × 0.170 × 0.205 mm) was obtained by evaporation from MeOH, separated from the sample, and mounted on a glass fiber, and data were collected using Incoatec microsource 3.0 Cu Kα radiation, λ = 0.710 73 Å at 150(2) K. Crystal data: C_14_H_14_N_2_O_3_, M = 258.27 g/mol, space group monoclinic, *P*21/*n*; unit cell dimensions *a* = 13.4585(7) Å, *b* = 7.6654(4) Å, *c* = 13.5148(8) Å, *V* = 1218.13(12) Å^3^, *Z* = 4, *D*_calcd_ = 1.408 g/cm^3^, μ = 0.101 mm^–1^, *F*(000) = 544. A total of 3986 frames were collected. The total exposure time was 21.12 h. The frames were integrated with the Bruker SAINT software package using a narrow-frame algorithm. The integration of the data using a monoclinic unit cell yielded a total of 22 030 reflections to a maximum θ angle of 26.39° (0.80 Å resolution), of which 2498 were independent (average redundancy 8.819, completeness = 99.9%, *R*_int_ = 3.98%, *R*_sig_ = 2.22%) and 2261 (90.51%) were greater than 2σ(*F*^2^). Data were corrected for absorption effects using the multi-scan method (SADABS). The ratio of minimum to maximum apparent transmission was 0.938. The calculated minimum and maximum transmission coefficients (based on crystal size) are 0.9800 and 0.9970. The structure was solved and refined using the Bruker SHELXTL software package. The final anisotropic full-matrix least-squares refinement on *F*^2^ with 228 variables converged at *R*_1_ = 3.29% for the observed data and *wR*_2_ = 8.83% [*I* > 2σ(*I*)] for all data. Crystallographic data for **6** have been deposited with the Cambridge Crystallographic Data Centre (CCDC deposition number 2060794).^20^

### Bioassays

Petri plate antifungal assays against *Candida albicans* (ATCC 10231) and *Cryptococcus neoformans* (H99) and antibacterial assays against *Staphylococcus aureus* (ATCC 29213) were conducted using procedures reported previously.^21,22^ Briefly, 100 mL of yeast malt agar and tryptic soy agar (Difco) were prepared, sterilized by autoclaving, and cooled to 45 °C. One to three milliliters of the inoculum suspension was transferred to the warm fluid and mixed thoroughly by gentle swirling to avoid bubbles. Then the agar was poured into Petri plates (100 × 15 mm; 5 mL each). In conducting the disk diffusion assay, each filter paper disk (6.25 mm in diameter) was impregnated with the sample to be tested (50 μg/disk). After evaporation of the solvent, the disk was placed on the agar surface and incubated at room temperature for 24–72 h. The antifungal agent amphotericin and the antibiotic gentamicin (Sigma Chemical Co.) at 25 μg/disk were used as positive controls.

### Minimum Inhibitory Concentration (MIC)

To quantify the inhibitory concentrations of compound **1** against bacterial and fungal pathogens, MICs were measured using species-specific modifications to standard CLSI testing methods.^23,24^ Briefly, overnight cultures of *Staphylococcus aureus* ATCC 43300, *C. albicans* ATCC 10231, and *C. neoformans* H99 were sequentially diluted to OD 600 of 0.8 in PBS and 1000× fold in RPMI-1640 buffered with MOPS. The final cell suspension was incubated with serial dilutions of **1** ranging from 0.25 to 128 μM. Growth was assessed by adding 10% alamarBlue (Bio-Rad) followed by incubation for 24 h (*S. aureus* ATCC 43300 at 37 °C) or 48 h (*C. albicans* ATCC 10231 at 23 °C and *C. neoformans* H99 at both 23 and 37 °C). Chlortetracycline + streptomycin was the control for *S. aureus*, while amphotericin B was the control for fungal strains.
