# Supplementary material for: Broomeanamides: Cyclic Octapeptides from an Isolate of the Fungicolous Ascomycete Sphaerostilbella broomeana from India
Source: J Nat Prod. 2021 Jun 30;84(7):2028–34. doi: 10.1021/acs.jnatprod.1c00414 (PMC8314271; doi:10.1021/acs.jnatprod.1c00414)
Supplement: Supplementary file 1 — np1c00414_si_001.pdf [file np1c00414_si_001.pdf]

Broomeanamides: Cyclic Octapeptides from an Isolate of Fungicolous *Sphaerostilbella broomeana* (Hypocreales, Ascomycota) from India

Dulamini I. Ekanayake,[†] Bruno Perlatti,[‡] Dale C. Swenson,[1] Kadri Põldmaa,[§] Gerald F. Bills[‡] and James B. Gloer*[,†]

[†]Department of Chemistry, University of Iowa, Iowa City, IA 52242, United States

[‡]Texas Therapeutic Institute, The Brown Foundation Institute of Molecular Medicine, University of Texas Health Science Center, 1881 East Road, Houston, TX 77054, United States

[§]Department of Botany, Institute of Ecology and Earth Sciences, University of Tartu, Lai 40, EE-51005 Tartu, Estonia

List of Supporting Information

Figure S1. Isolate TFC201724 grown for 5d on MEA*;* a – colony, b–d conidiophores, e – conidia.

Figure S2. Maximum likelihood phylogeny of the *Sphaerostilbella broomeana*-group based on translation elongation factor (TEF; 1232 bp) and ITS-LSU (1461 bp) ribosomal DNA sequences[3]. *Sphaerostilbella parabroomeana* was used as an outgroup; bootstrap support values >95% indicated above the branches.

Figure S3. ^{1}H NMR Spectrum of Broomeanamide A (**1**; 600 MHz, $CDCl_3$)

Figure S4. ^{13}C NMR Spectrum of Broomeanamide A (**1**; 400 MHz, $CDCl_3$)

Figure S5. TOCSY Spectrum of Broomeanamide A (**1**; 600 MHz, $CDCl_3$)

Figure S6. HSQC Spectrum of Broomeanamide A (**1**; 600 MHz, $CDCl_3$)

Figure S7. HMBC Spectrum of Broomeanamide A (**1**; 600 MHz, $CDCl_3$)

Figure S8. Expanded Carbonyl Region of the HMBC Spectrum of Broomeanamide A (**1**; 600 MHz, $CDCl_3$)

Figure S9. ROESY Spectrum of Broomeanamide A (**1**; 600 MHz, $CDCl_3$)

Figure S10. HRESIMSMS data for Broomeanamide A (**1**)

Figure S11. HPLC-MS data from Marfey's analysis of **1**.

Figure S12. ^{1}H NMR Spectrum of Broomeanamide B (**2**; 500 MHz, $CDCl_3$)

Figure S13. TOCSY Spectrum of Broomeanamide B (**2**; 500 MHz, $CDCl_3$)

Figure S14. HSQC Spectrum of Broomeanamide B (**2**; 500 MHz, $CDCl_3$)

Figure S15. HMBC Spectrum of Broomeanamide B (**2**; 500 MHz, $CDCl_3$)

Figure S16. ^1H NMR Spectrum of Broomeanamide C (**3**; 500 MHz, $CDCl_3$)

Figure S17. TOCSY Spectrum of Broomeanamide C (**3**; 500 MHz, $CDCl_3$)

Figure S18. HSQC Spectrum of Broomeanamide C (**3**; 500 MHz, $CDCl_3$)

Figure S19. HMBC Spectrum of Broomeanamide C (**3**; 500 MHz, $CDCl_3$)

Figure S20. MIC data

Figure S21. ^1H NMR Spectrum of **4** (500 MHz, $CDCl_3$)

Figure S22. NOESY Spectrum of **4** (600 MHz, $CDCl_3$)

Figure S23. Expanded Aromatic Region of the NOESY Spectrum of **4** (**4**; 600 MHz, $CDCl_3$)

Figure S24. ^1H NMR Spectrum of **5** (600 MHz, $CDCl_3$)

Figure S25. ^{13}C NMR Spectrum of **5** (100 MHz, $CDCl_3$)

Figure S26. ^1H NMR Spectrum of **6** (600 MHz, $CDCl_3$)

Figure S27. ^{13}C NMR Spectrum of **6** (100 MHz, $CDCl_3$)

Figure S28. ^1H NMR Spectrum of **7** (600 MHz, $CDCl_3$)

Table S1. NMR spectroscopic data of broomeanamide B (**2**) in $CDCl_3$

Table S2. NMR spectroscopic data of broomeanamide C (**3**) in $CDCl_3$

*To whom correspondence should be addressed. Tel: 319-335-1361. E-mail: james-gloer@uiowa.edu.

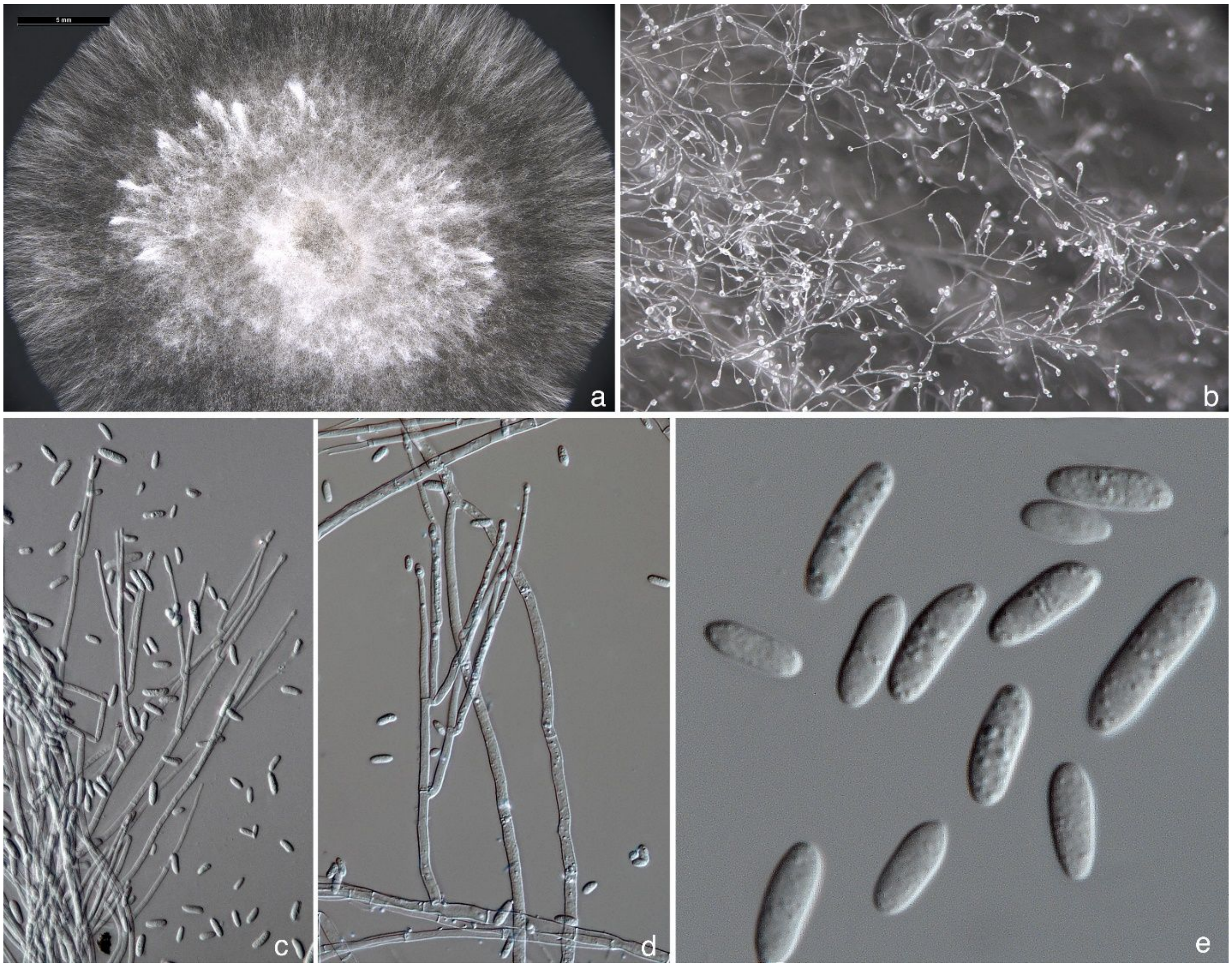

Figure S1. Isolate TFC201724 grown for 5d on MEA*;* a – colony, b–d conidiophores, e – conidia.

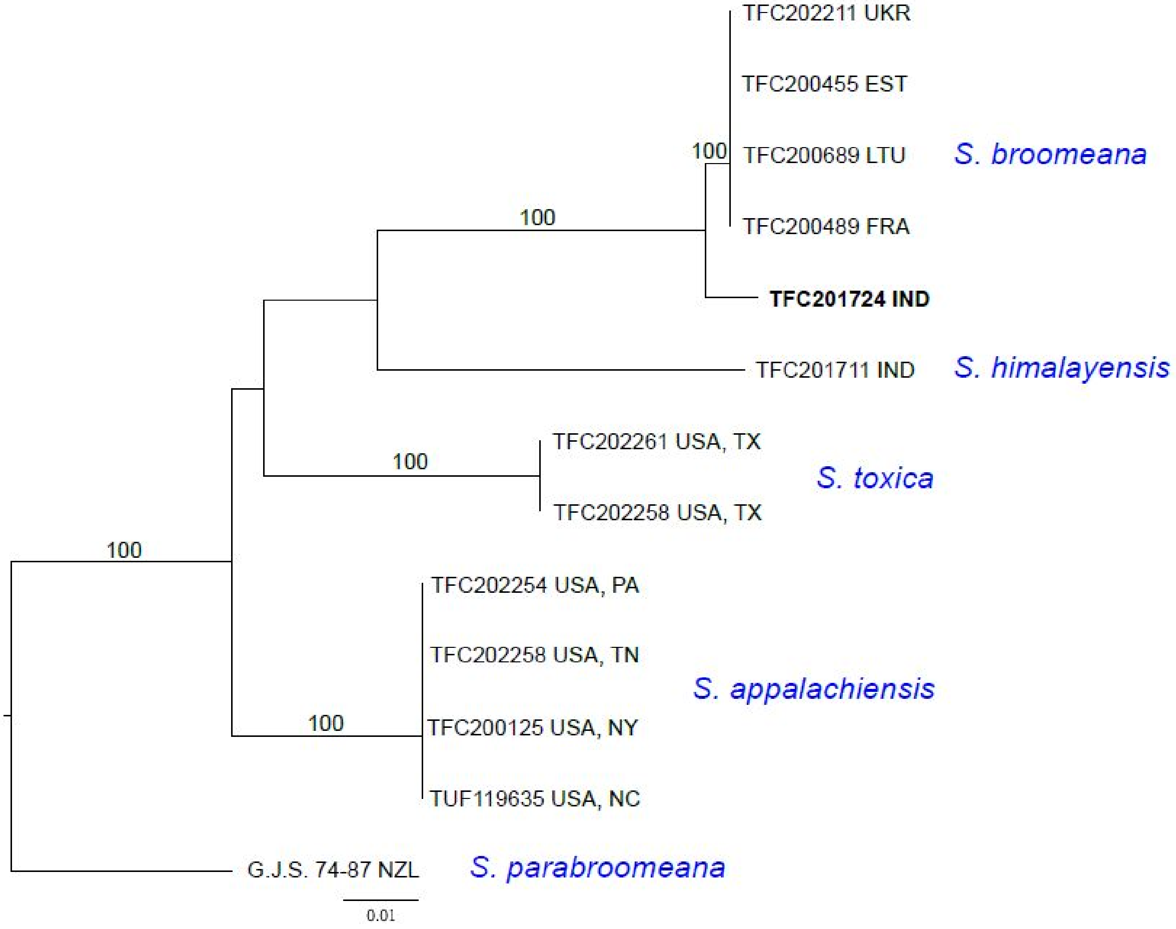

Figure S2. Maximum likelihood phylogeny of the *Sphaerostilbella broomeana*-group based on translation elongation factor (TEF; 1232 bp) and ITS-LSU (1461 bp) ribosomal DNA sequences[3]. *Sphaerostilbella parabroomeana* was used as an outgroup; bootstrap support values >95%

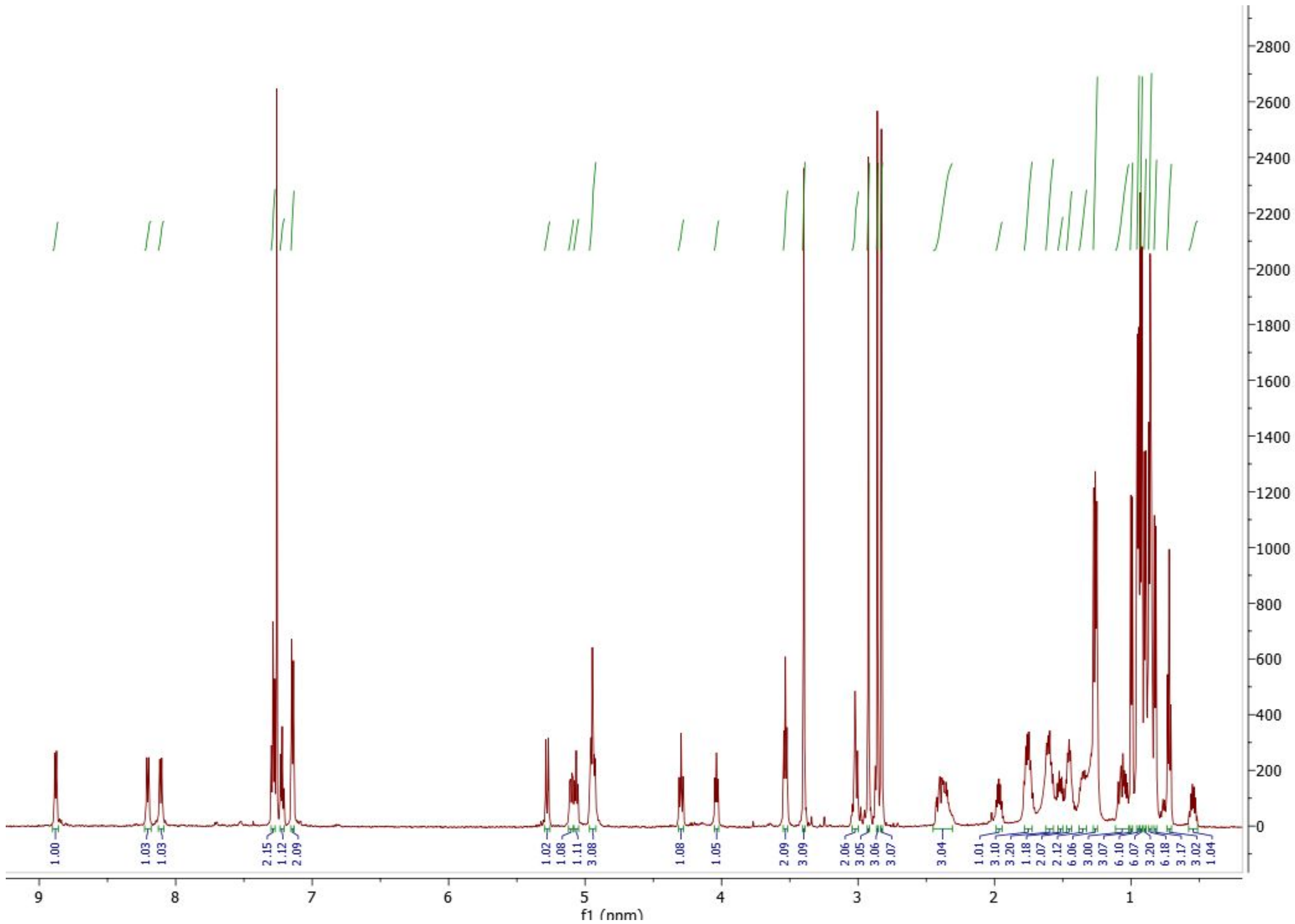

Figure S3. ^{1}H NMR Spectrum of Broomeanamid

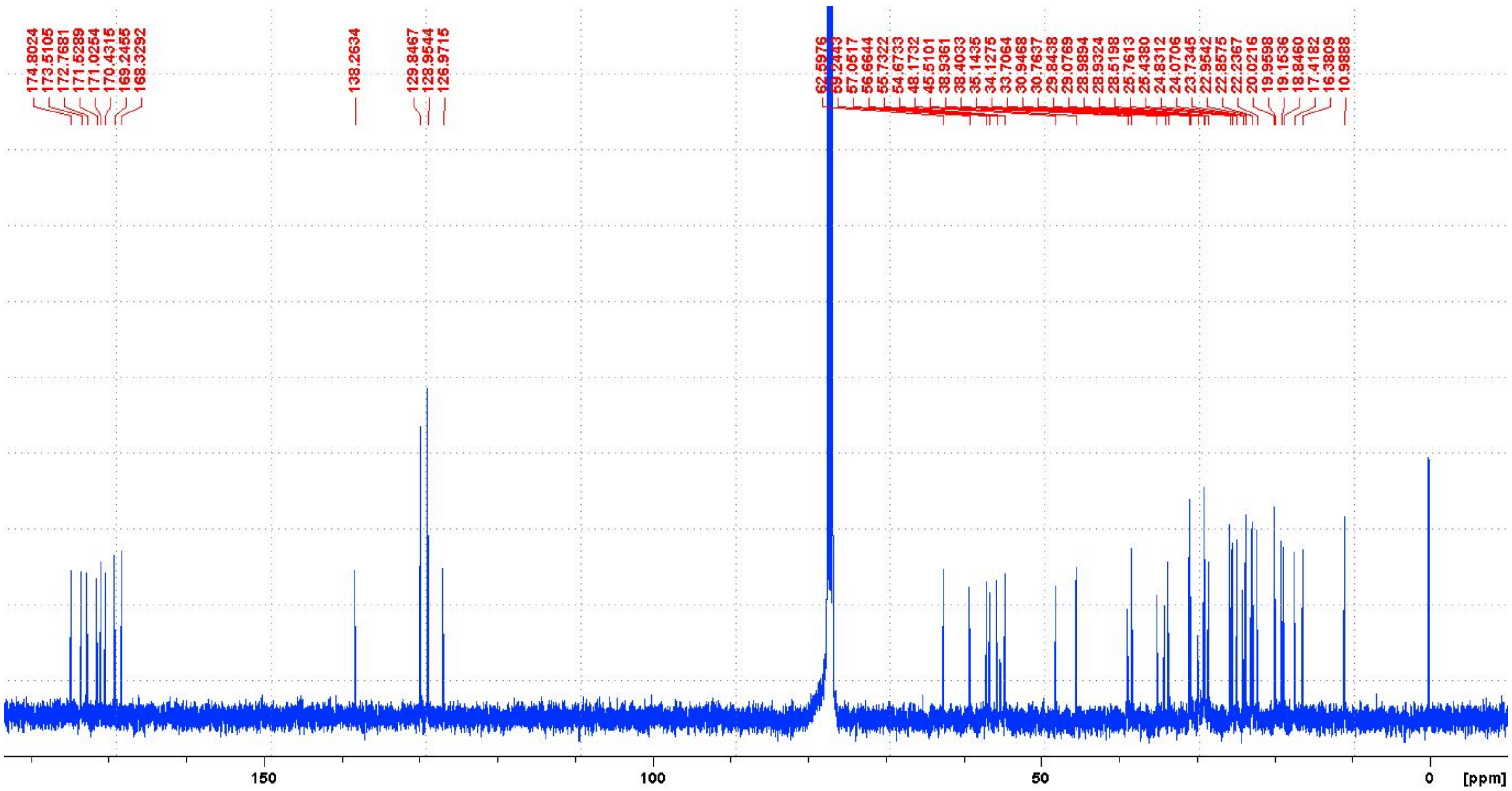

Figure S4. ^{13}C NMR Spectrum of Broomeanamide A (**1**

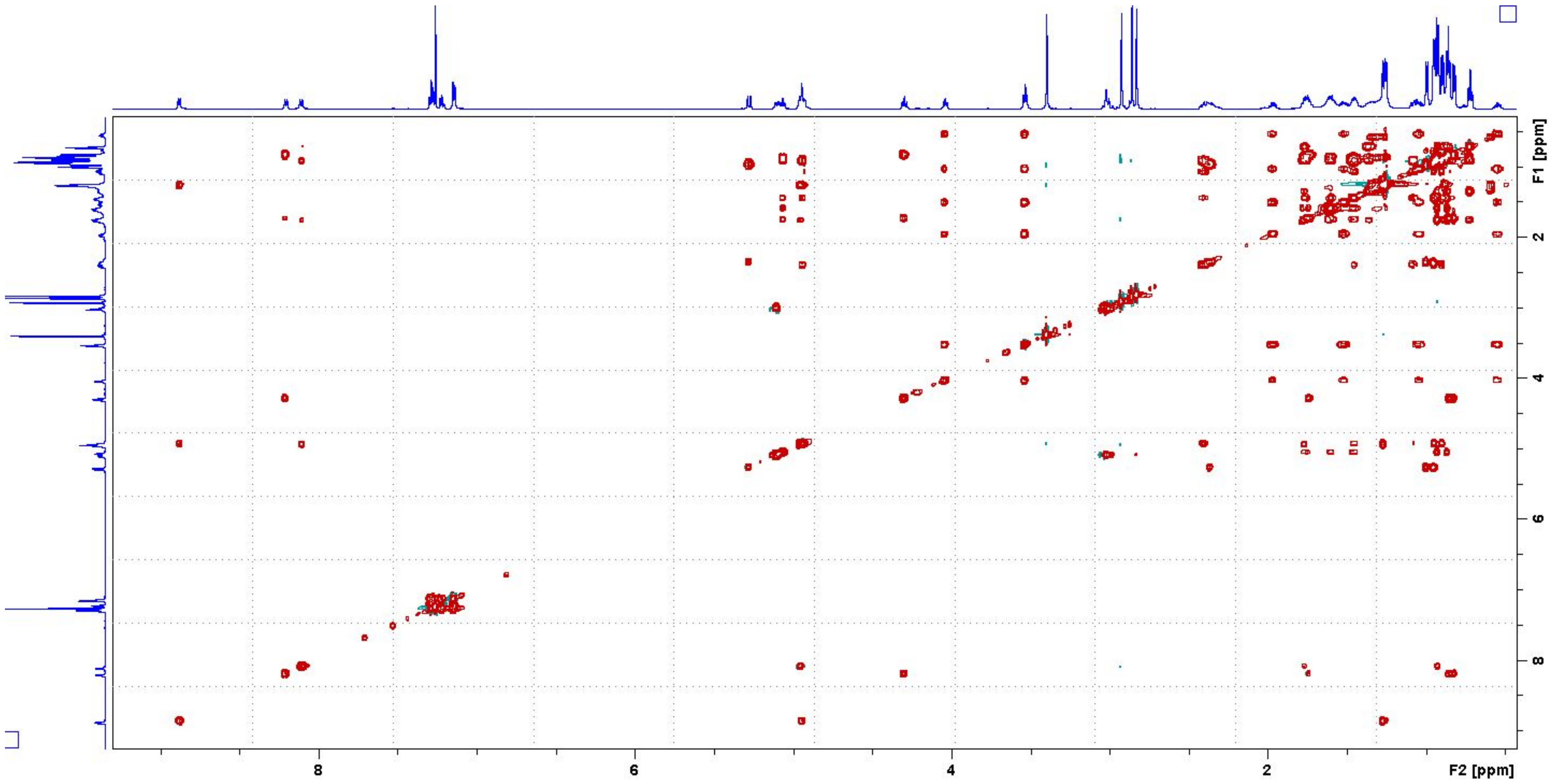

Figure S5. TOCSY Spectrum of Broomeanamide A (**1**; 6

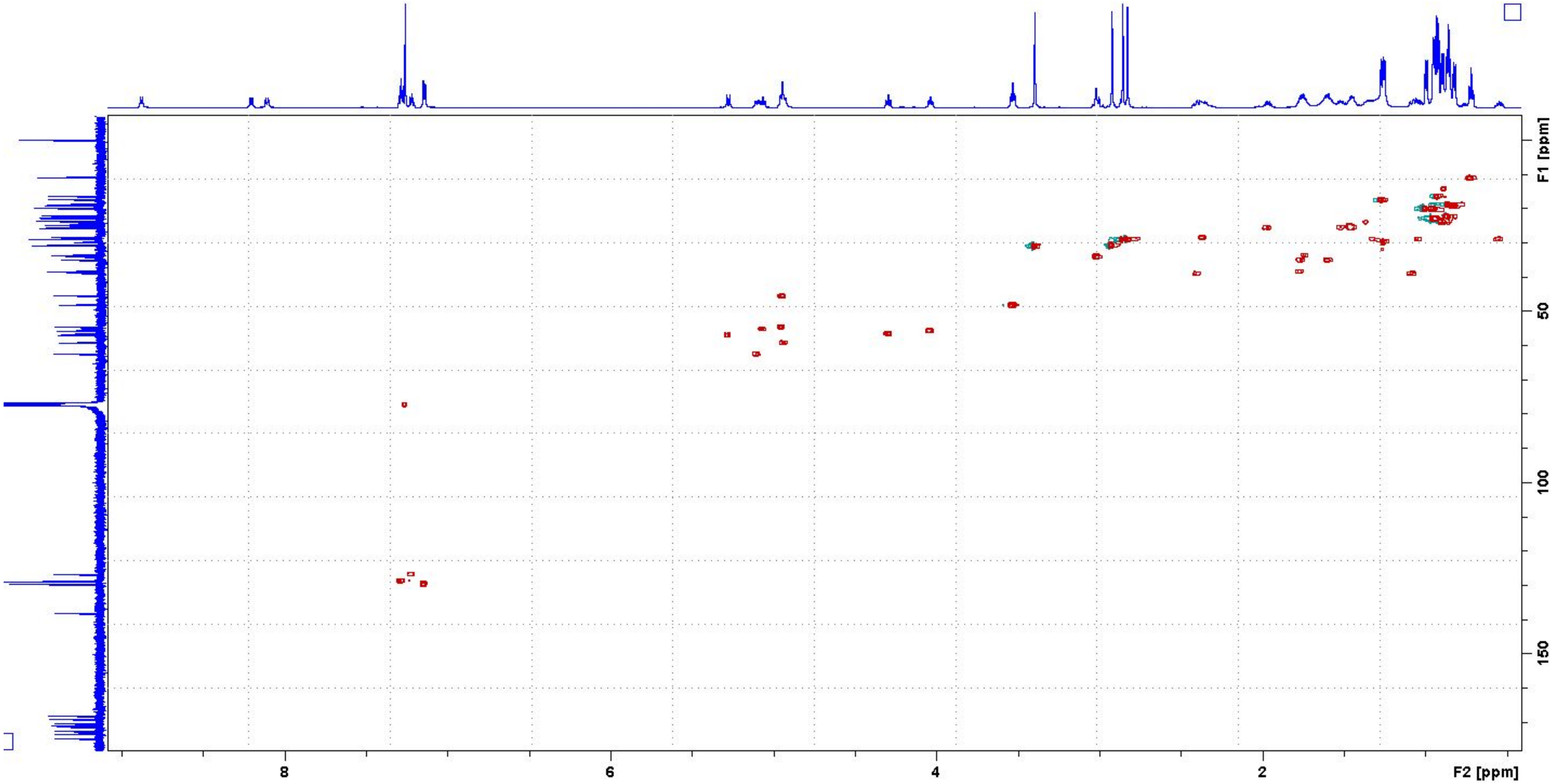

Figure S6. HSQC Spectrum of Broomeanamide A (**1**; 600

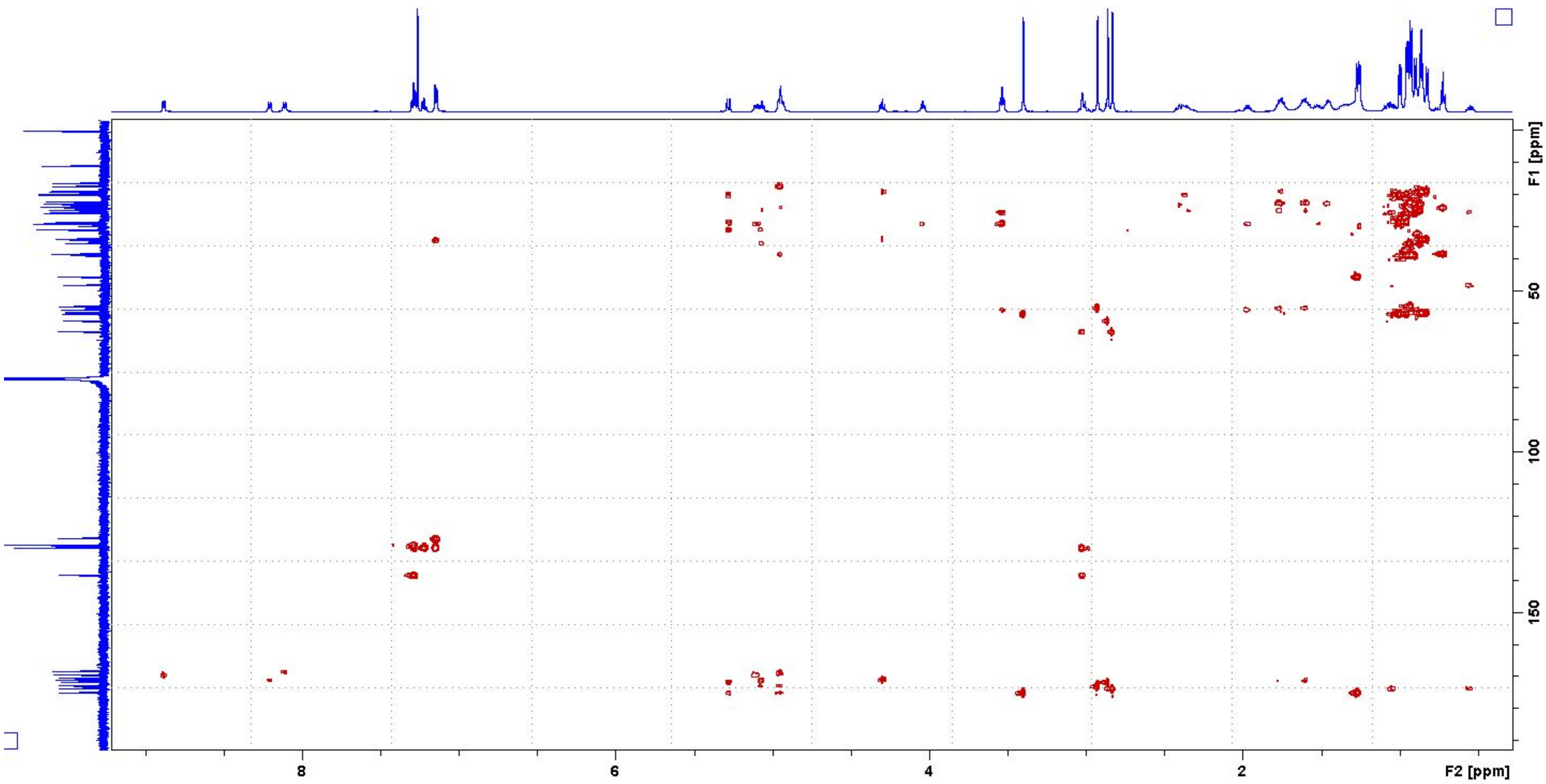

Figure S7. HMBC Spectrum of Broomeanamide A (**1**; 60

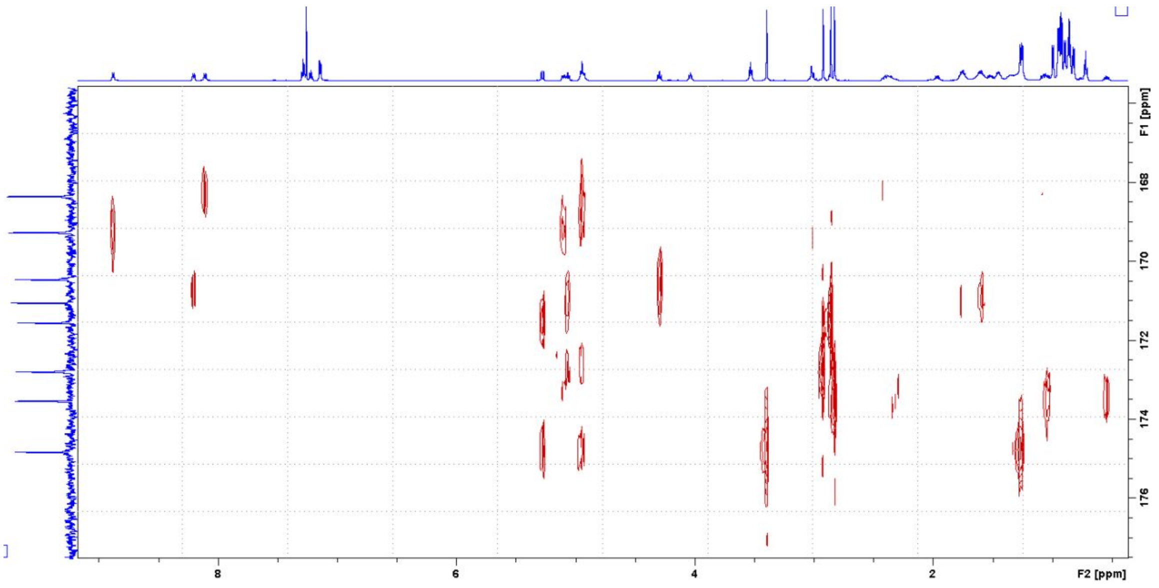

Figure S8. Expanded Carbonyl Region of the HMBC Spectrum of Broomeanamide A (**1**; 600 MHz, $CDCl_3$)

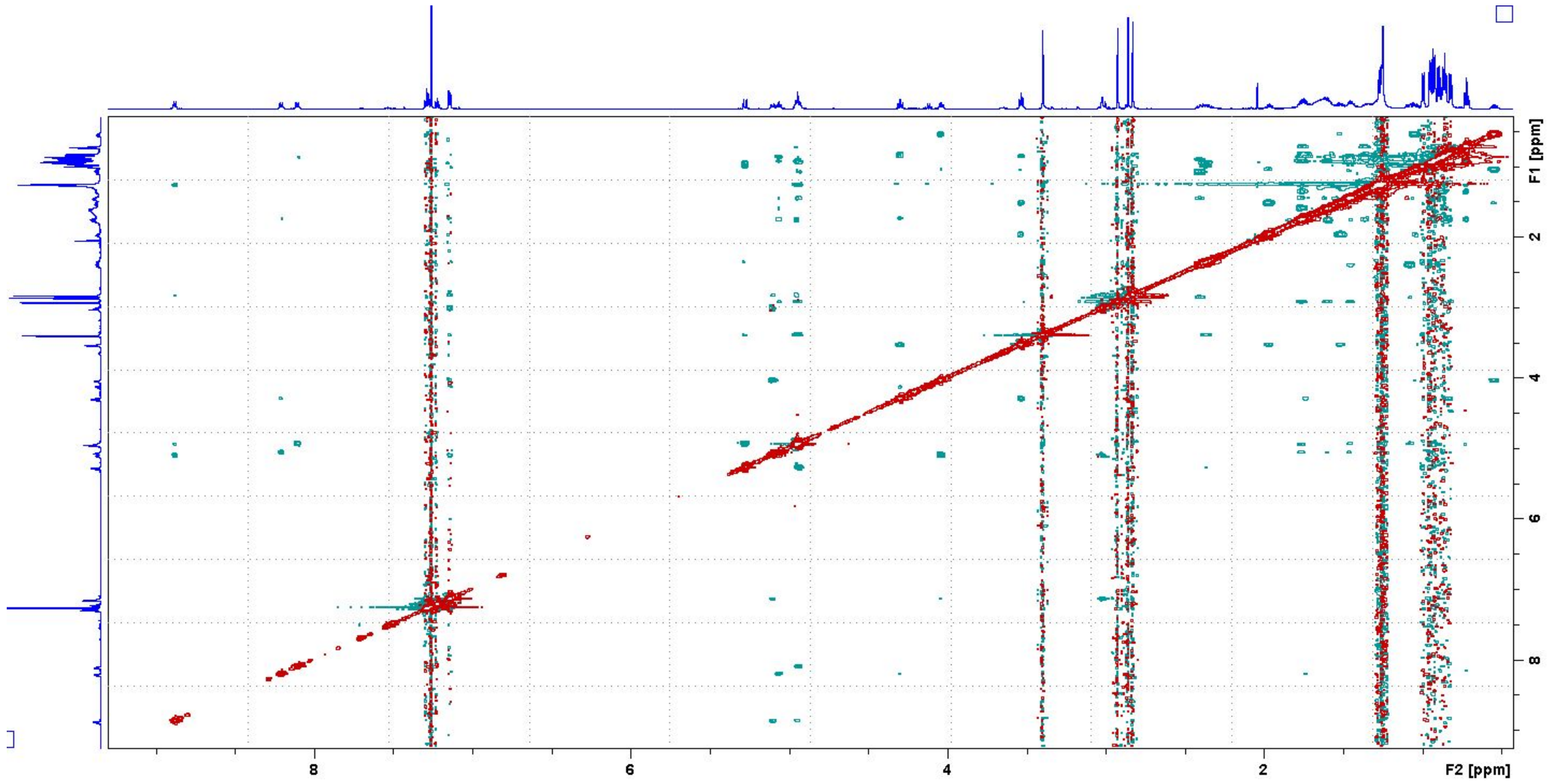

Figure S9. ROESY Spectrum of Broomeanamide A (**1**; 6

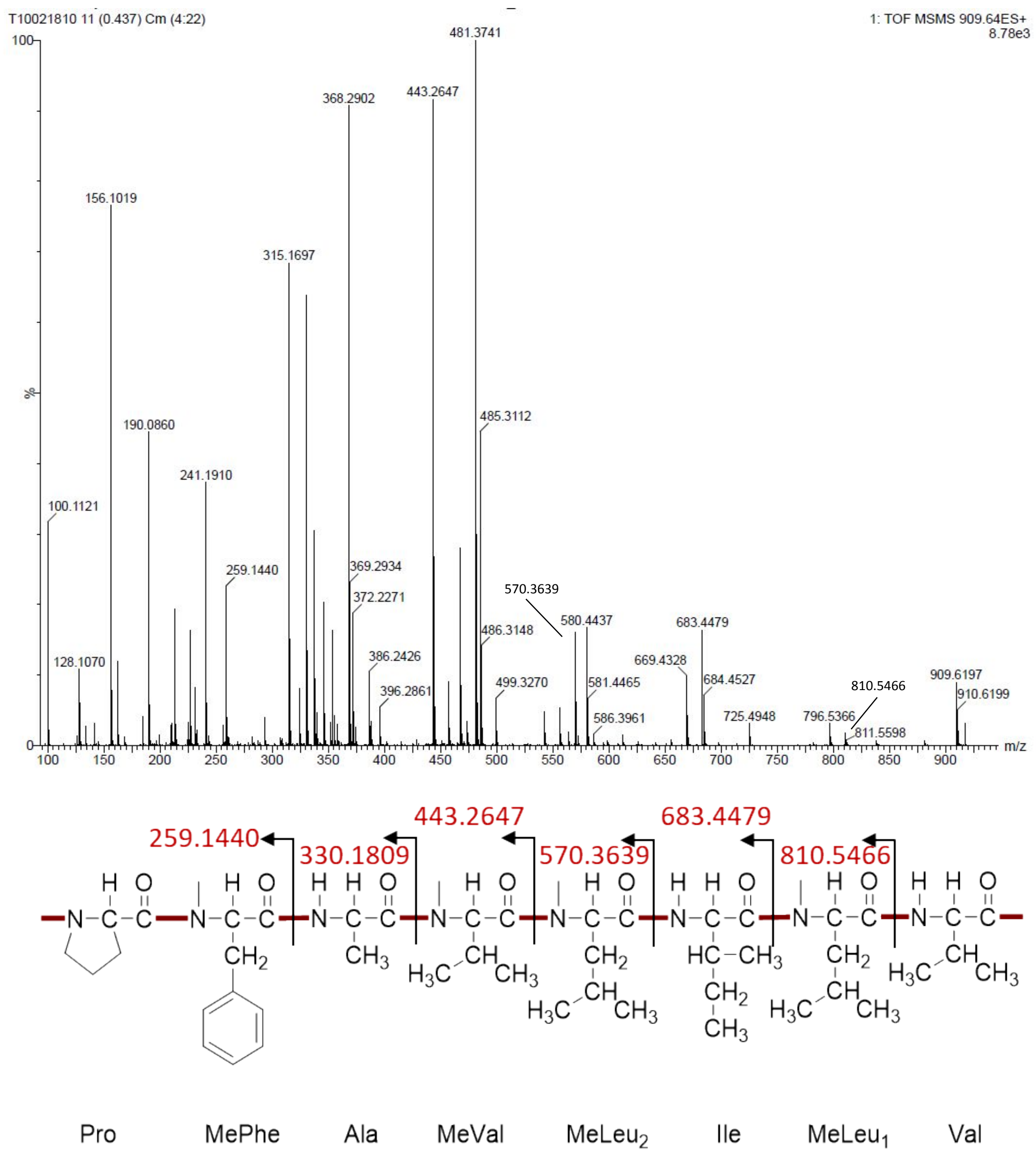

Figure S10. HRESIMSMS data for Broomeanamide A (**1**)

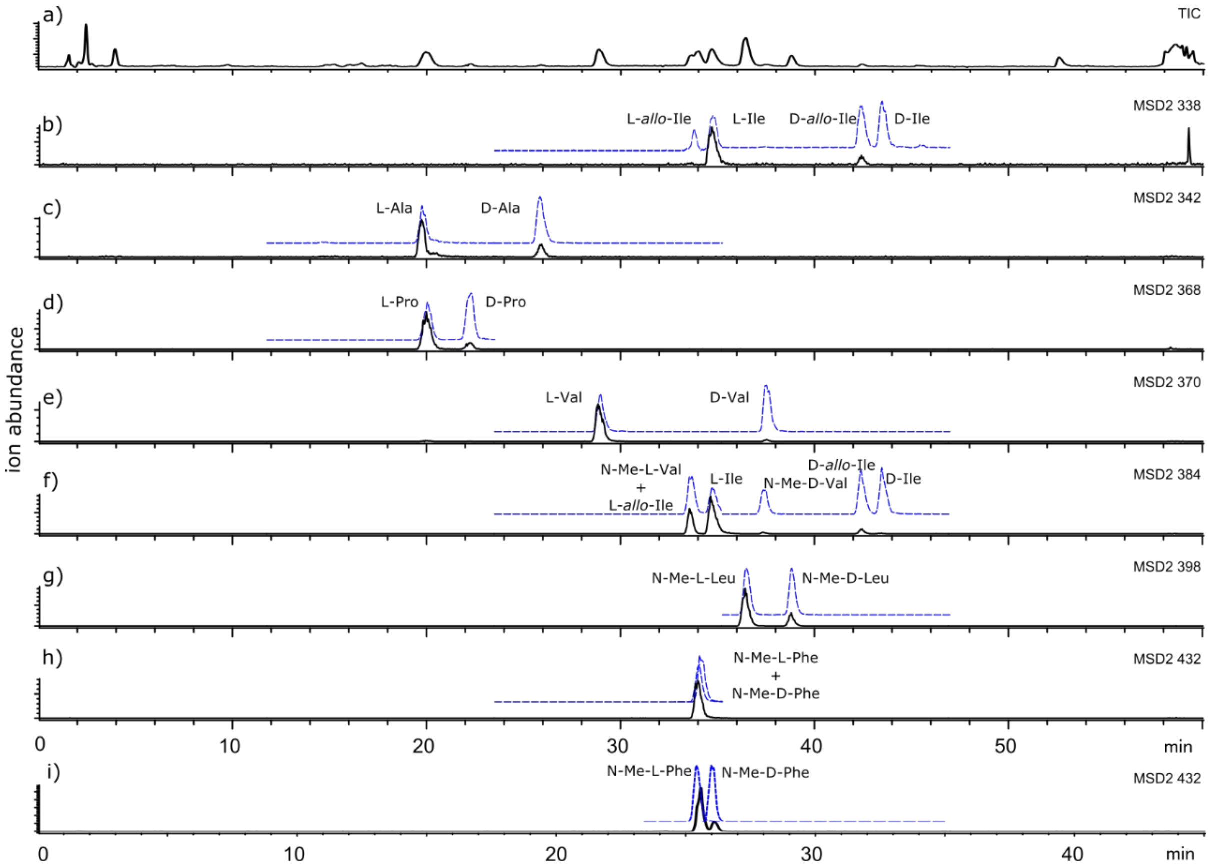

a) Total Ion Chromatogram using C_3 column; b) EIC (extracted ion current; *m/z* 338) for L-FDAA-L-Ile; c) EIC (*m/z* 342) for L-FDAA-L-Ala; d) EIC (*m/z* 368) for L-FDAA-L-Pro; e) EIC (*m/z* 370) for L-FDAA-L-Val; f) EIC (*m/z* 384) for L-FDAA-N-Me-L-Val (note the co-extraction of ions for the isomeric Ile residues); g) EIC (*m/z* 396) for L-FDAA-N-Me-L-Leu; h) EIC (*m/z* 432) for L-FDAA-N-Me-L-Phe; i) EIC spectrum (*m/z* 432) from C_{18} Marfey's analysis of **1** for characterization of L-FDAA-N-Me-L-Phe. For b)-i), L-FDAA derivatives of D- and L-amino acid standards are displayed as blue dotted lines.

Figure S11. HPLC-MS data from Marfey's analysis of **1**.

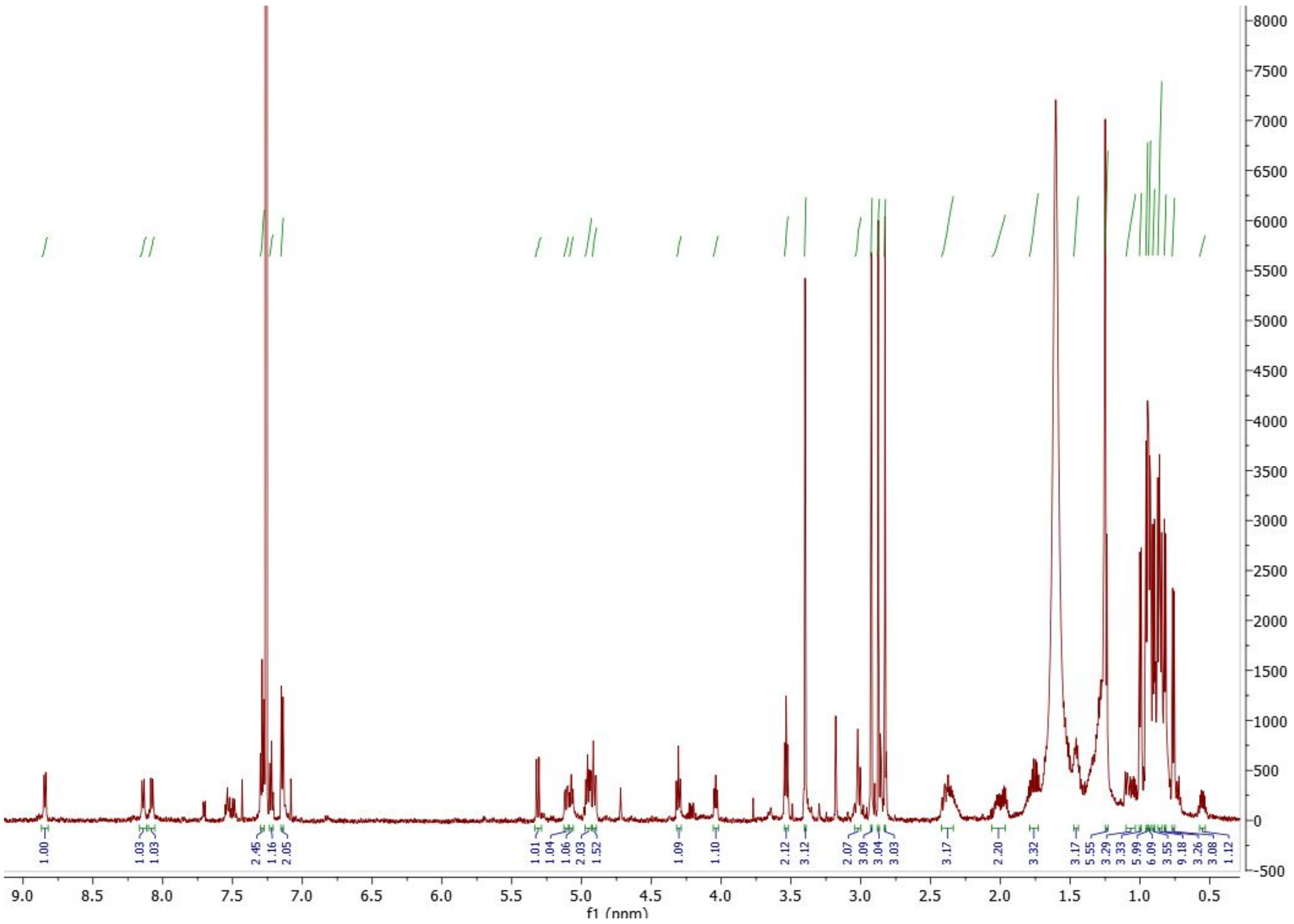

Figure S12. ^{1}H NMR Spectrum of Broome[illegible]

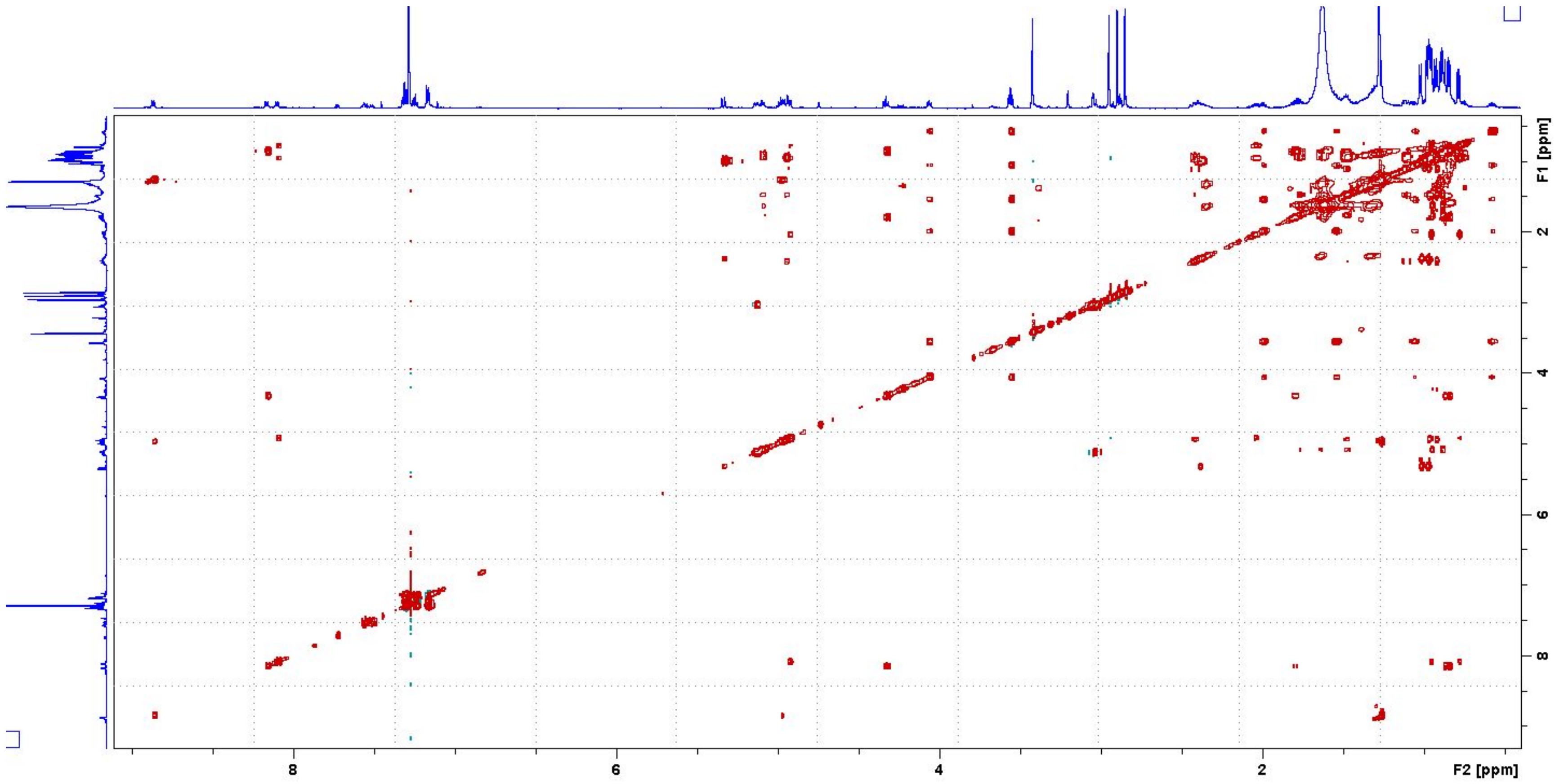

Figure S13. TOCSY Spectrum of Broomeanamide B (**2**;

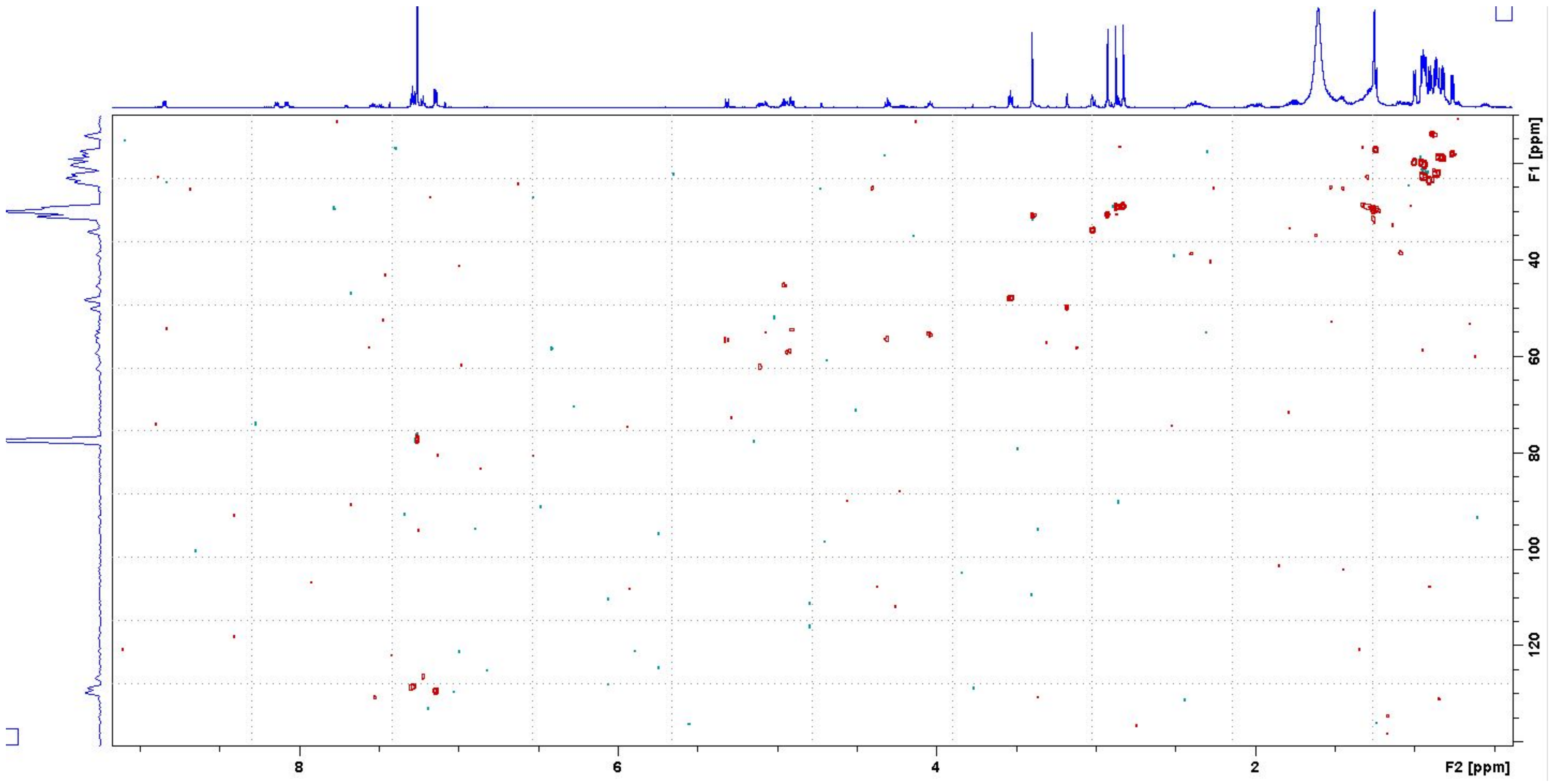

Figure S14. HSQC Spectrum of Broomeanamide B (**2**; 5

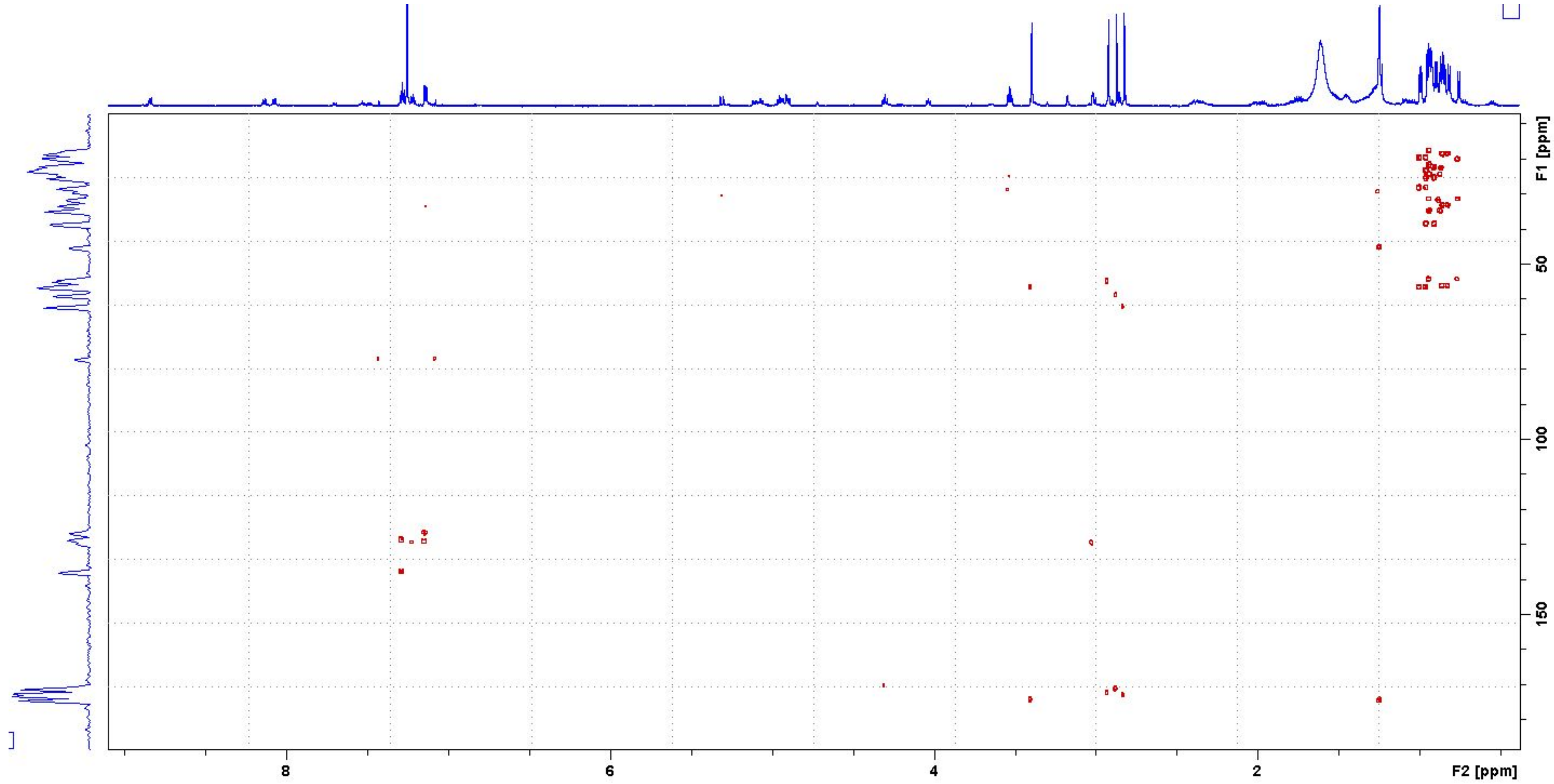

Figure S15. HMBC Spectrum of Broomeanamide B (**2**; [illegible]

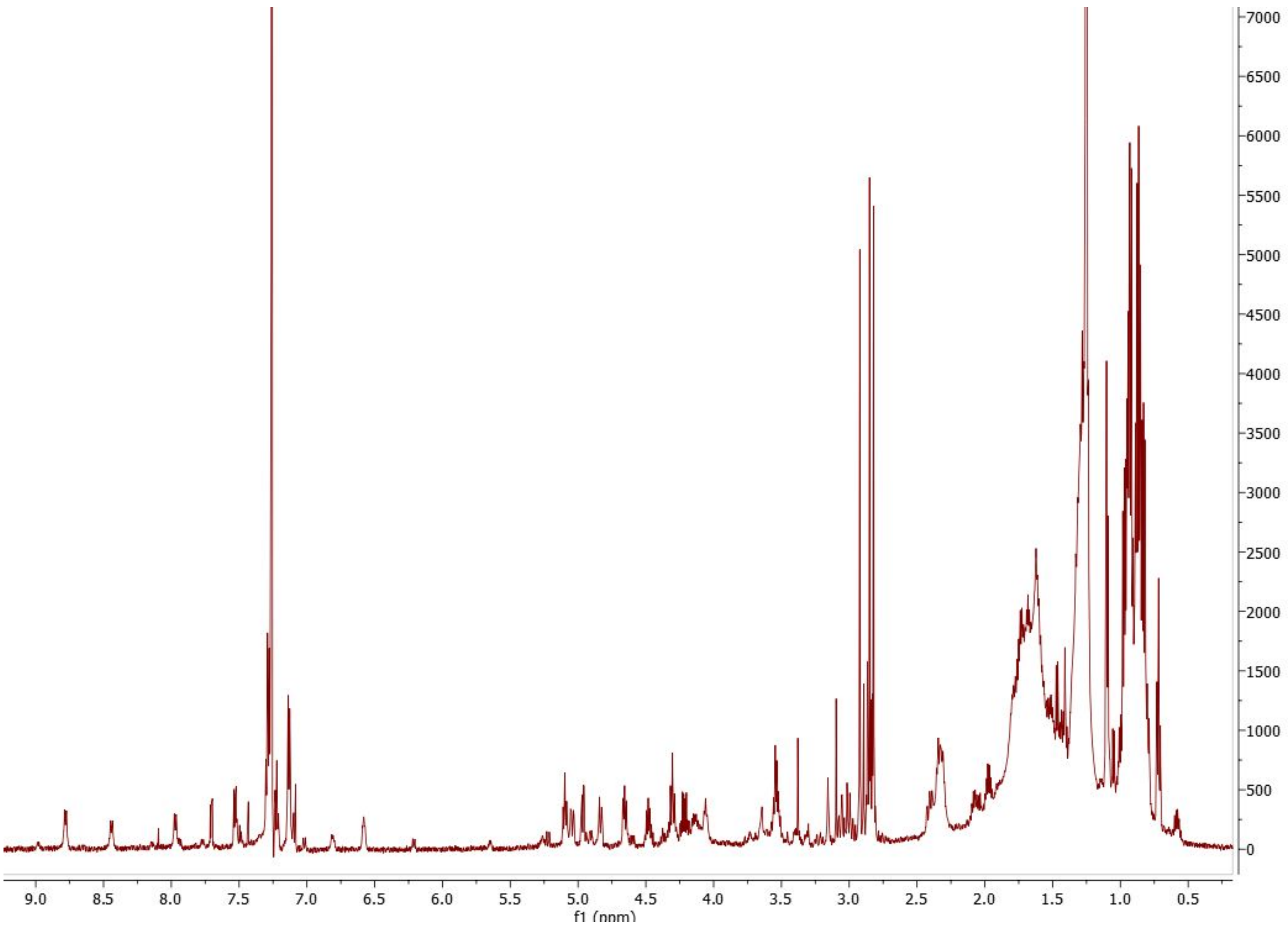

Figure S16. ^{1}H NMR Spectrum of Broomea

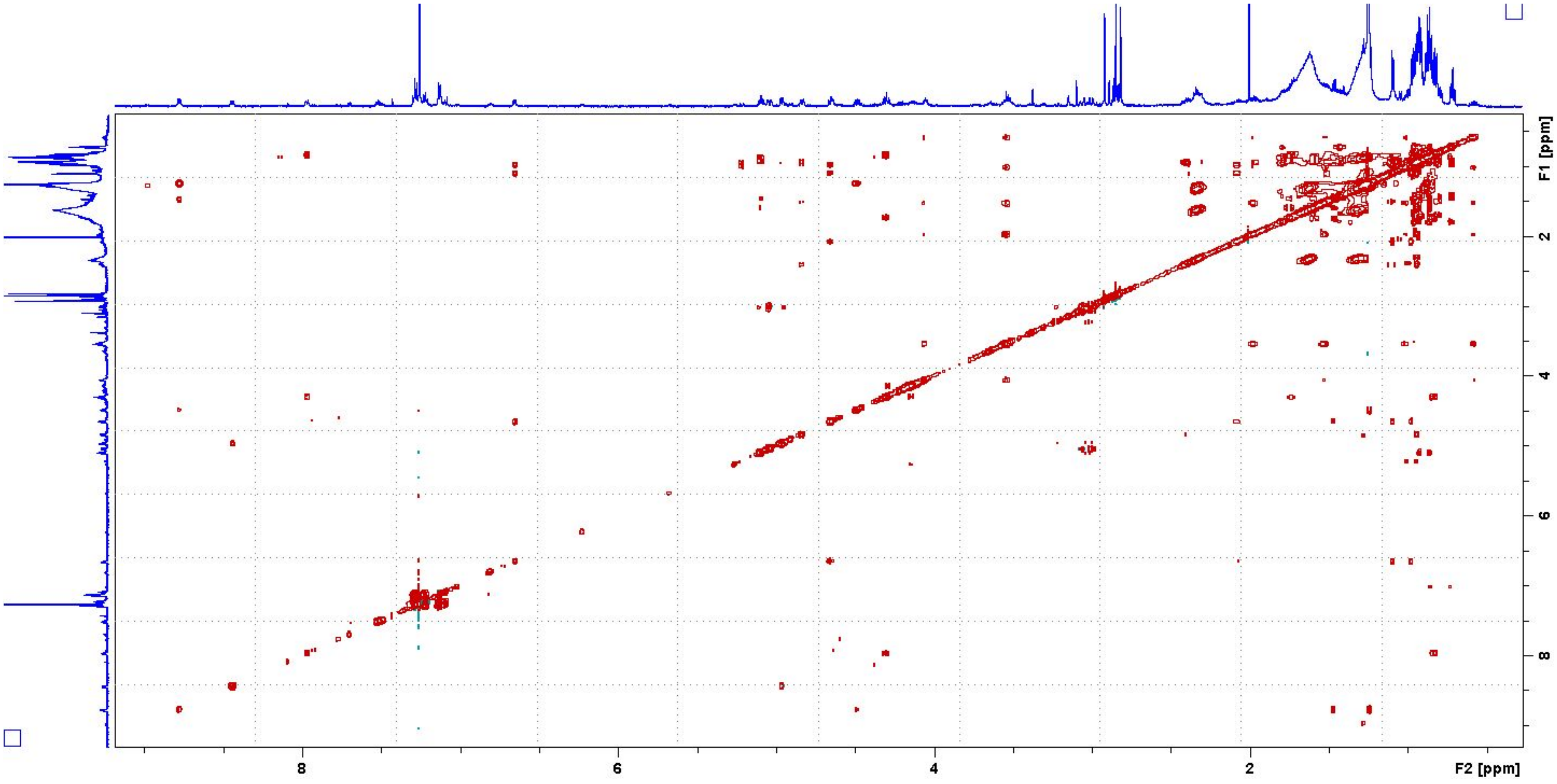

Figure S17. TOCSY Spectrum of Broomeanamide C (**3**;

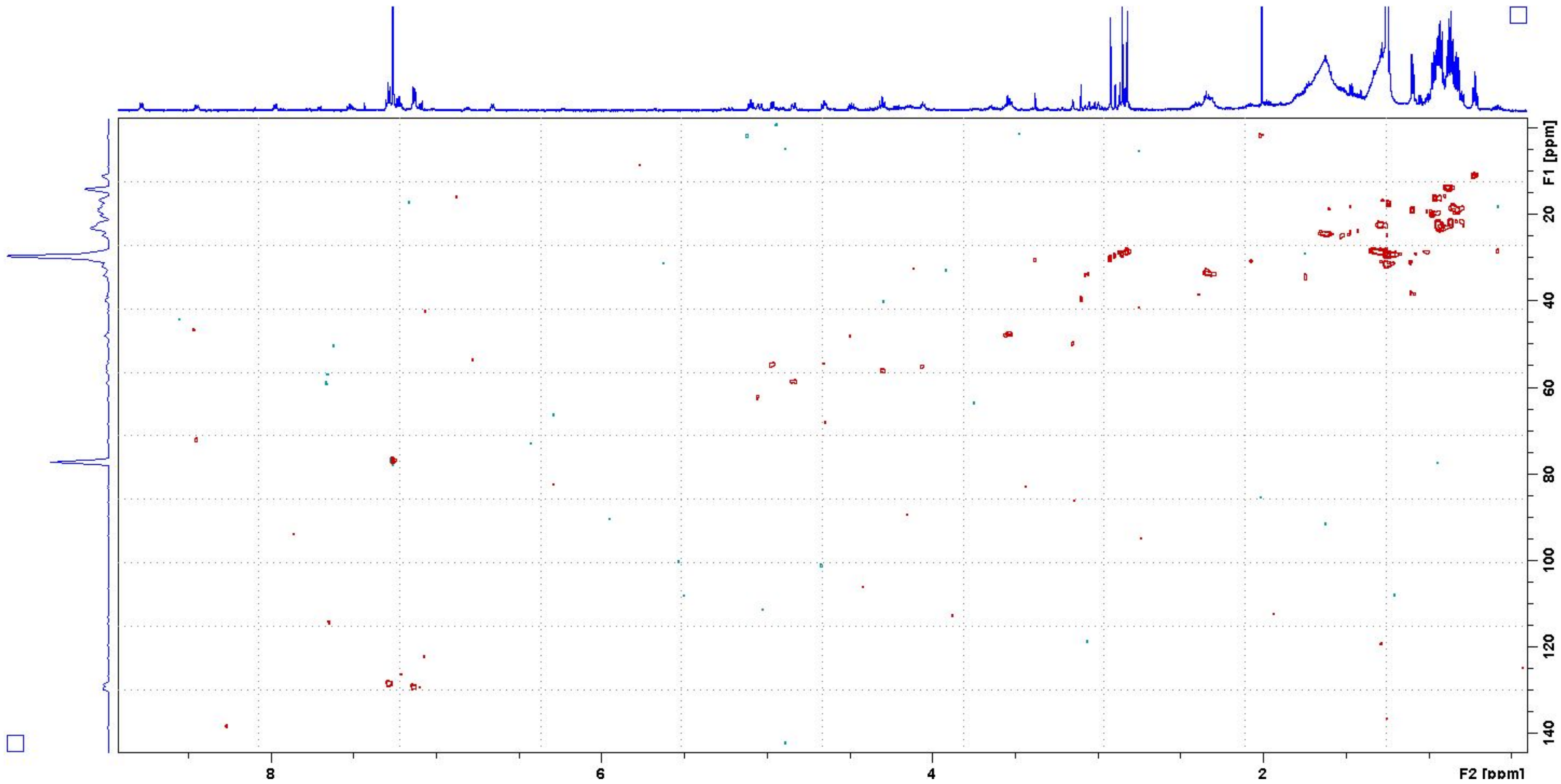

Figure S18. HSQC Spectrum of Broomeanamide C (**3**; 5[illegible]

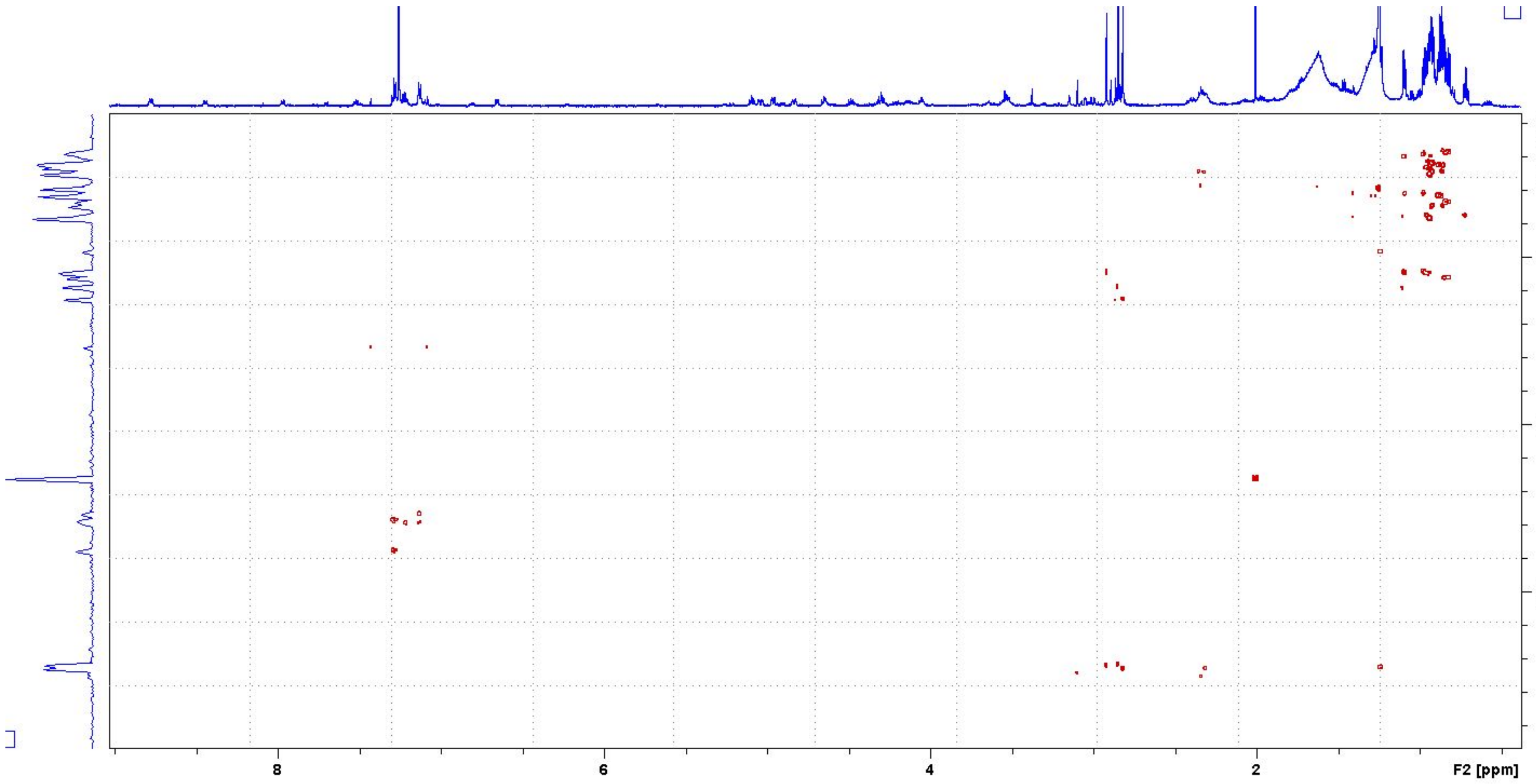

Figure S19. HMBC Spectrum of Broomeanamide C (**3**; [illegible]

***S. aureus* 23°C** ***C. albicans* 23°C**

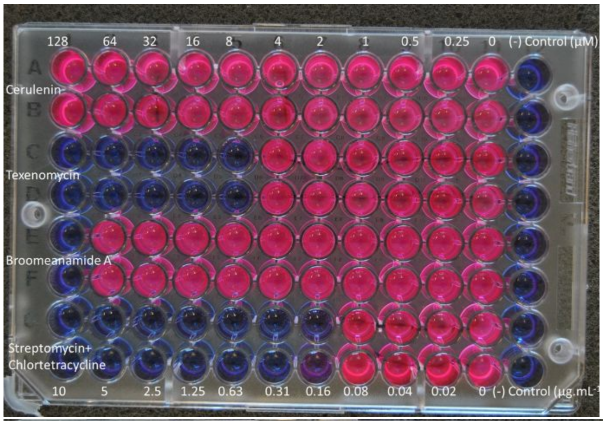

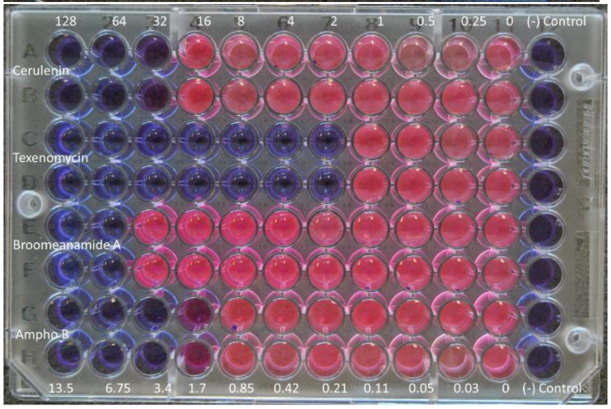

***C. neoformans* 23°C** ***C. neoformans* 37°C**

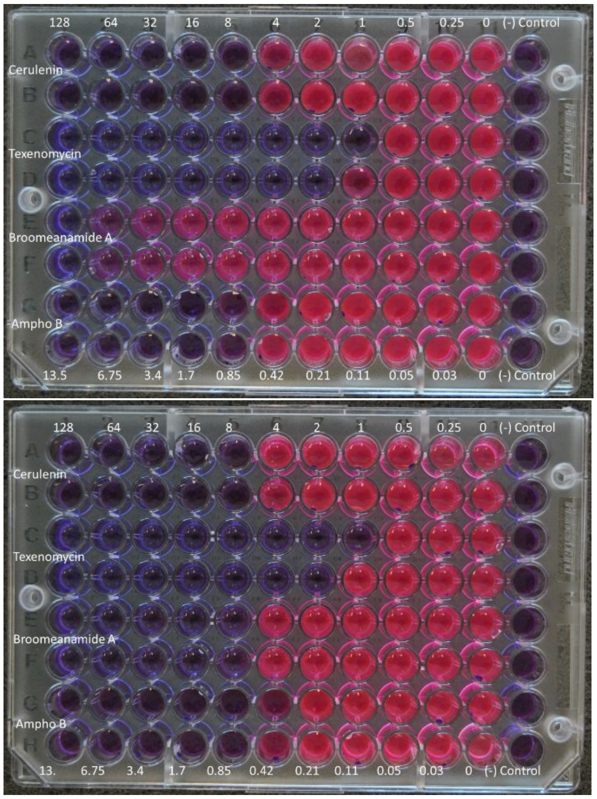

Figure S20. MIC data

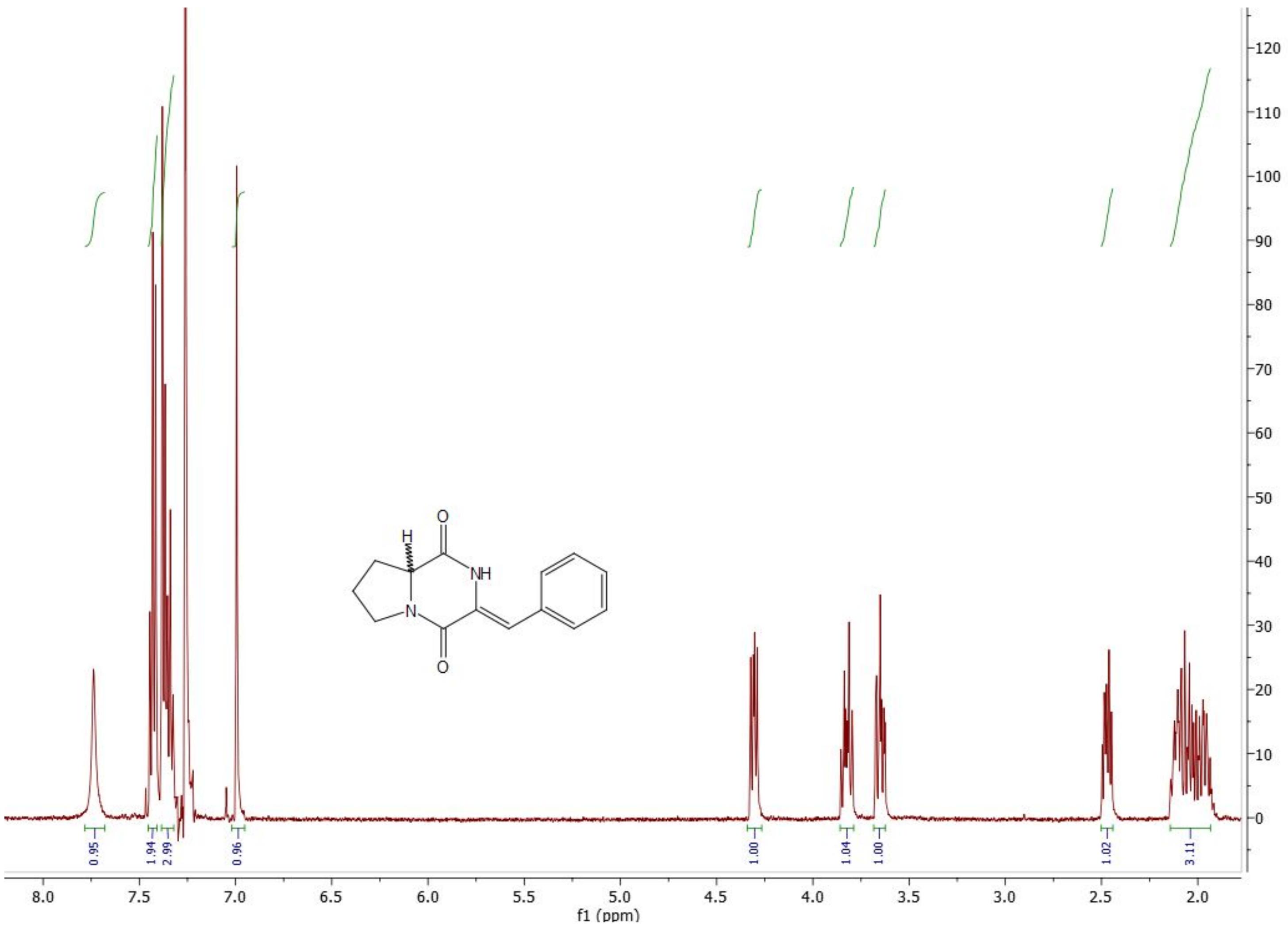

Figure S21. ^{1}H NMR Spectrum of **4** (600 MHz, CDC

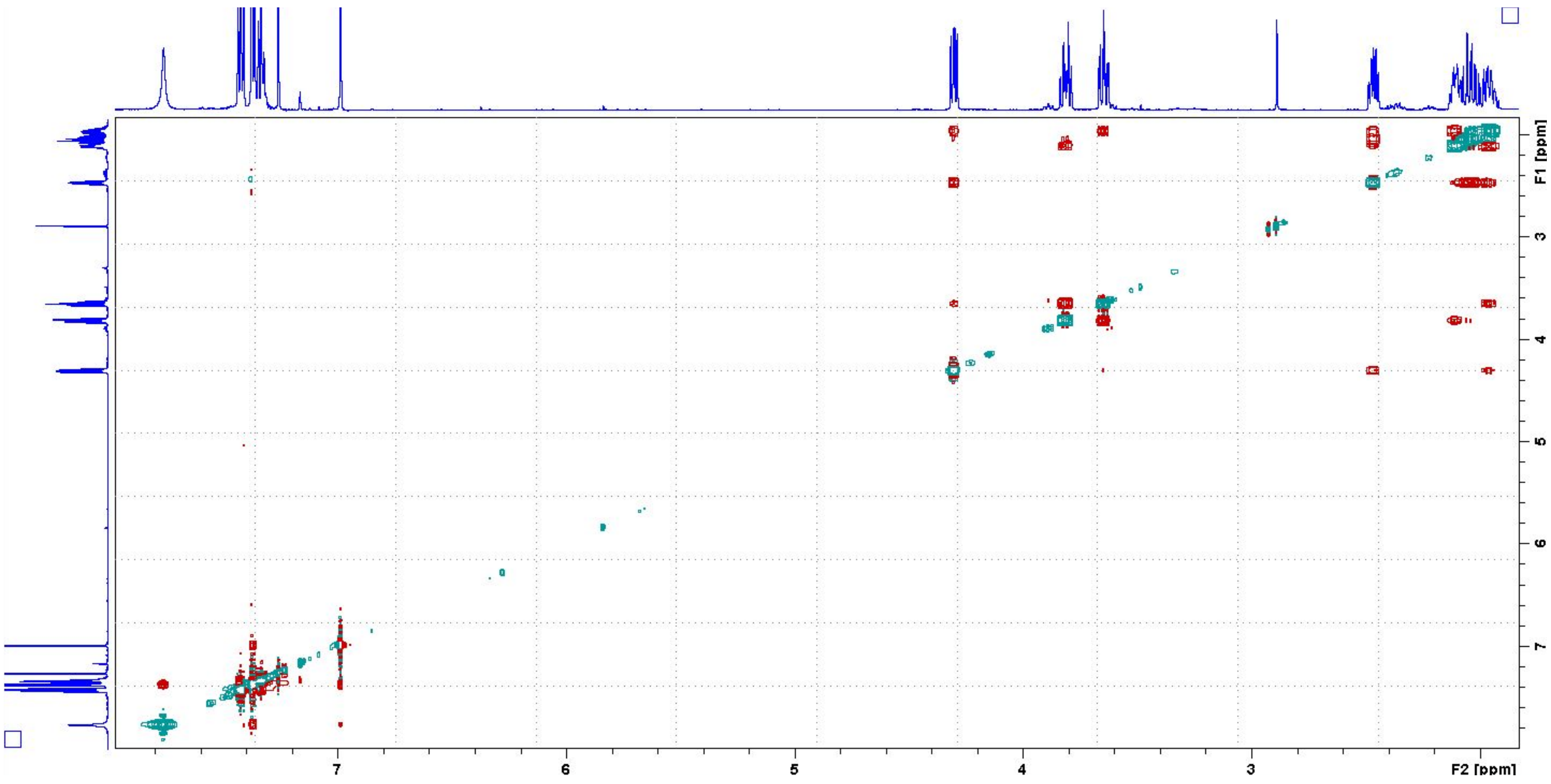

Figure S22. NOESY Spectrum of **4** (150 MHz, $CDCl_3$)

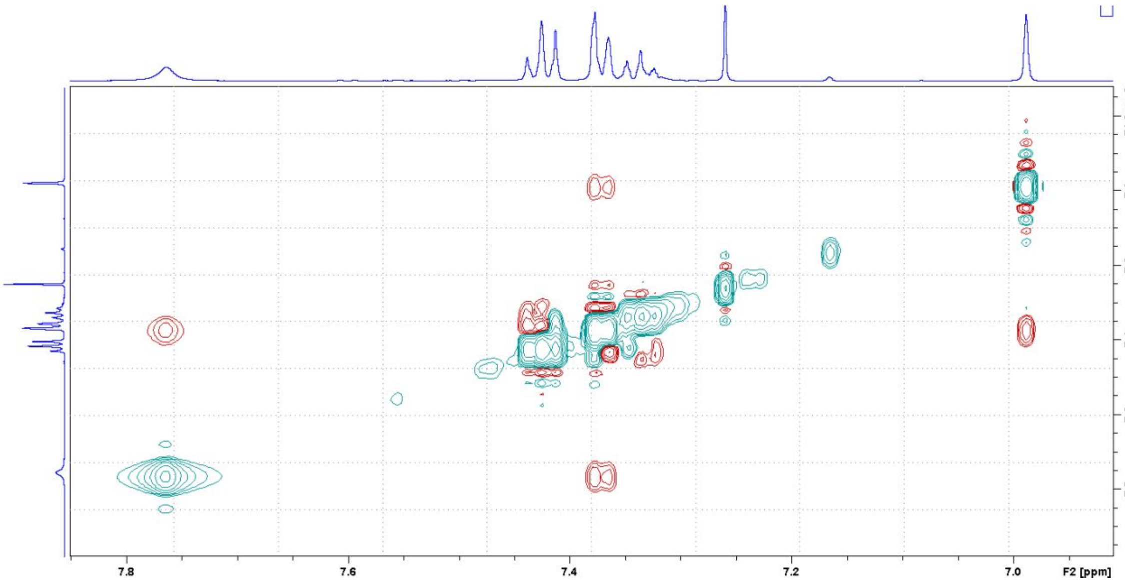

Figure S23. Expanded Aromatic Region of the NOESY Spectrum of **4** (**4**; 600 MHz, $CDCl_3$)

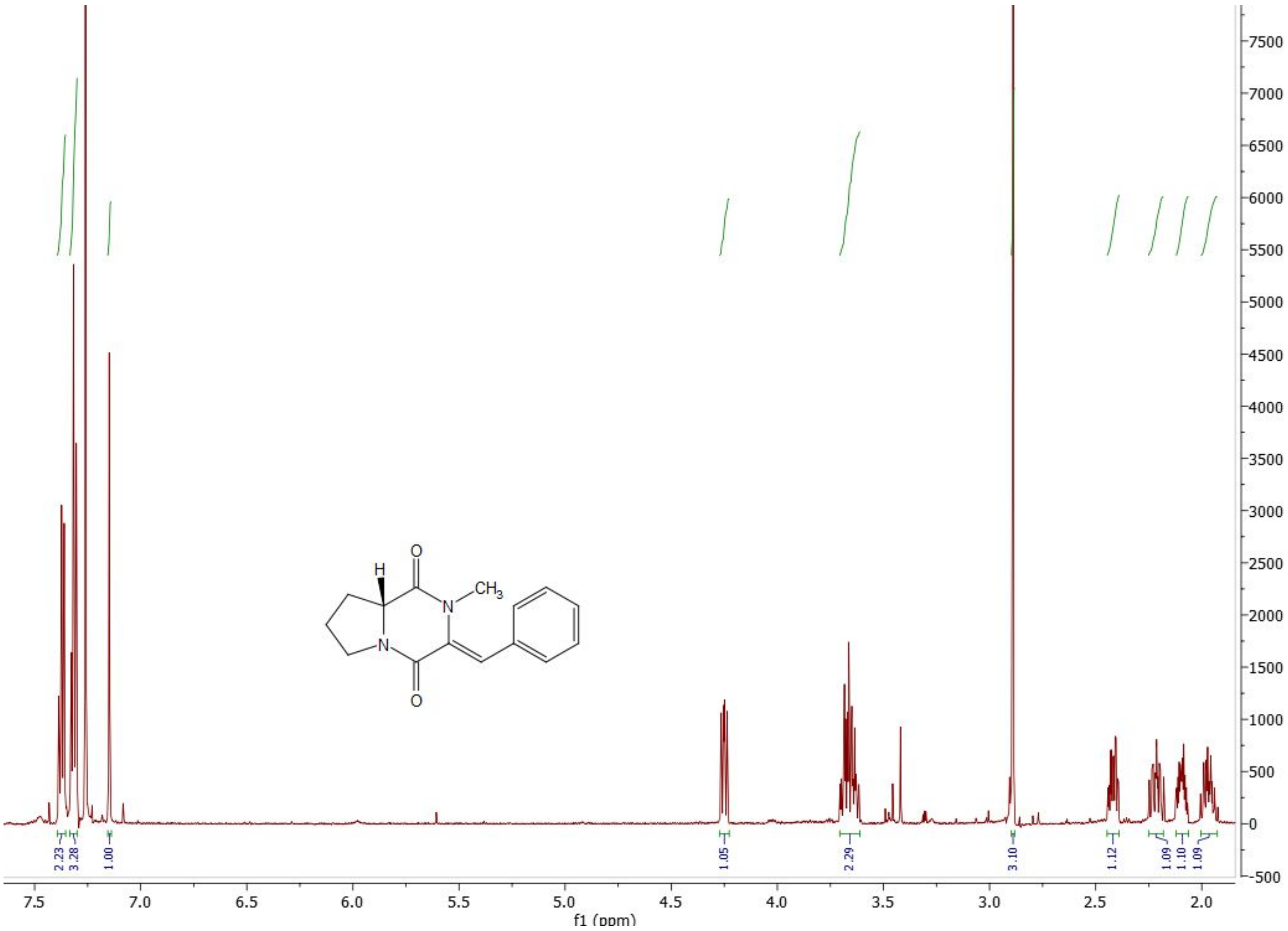

Figure S24. ^{1}H NMR Spectrum of **5** (600 MHz, $CDCl_3$)

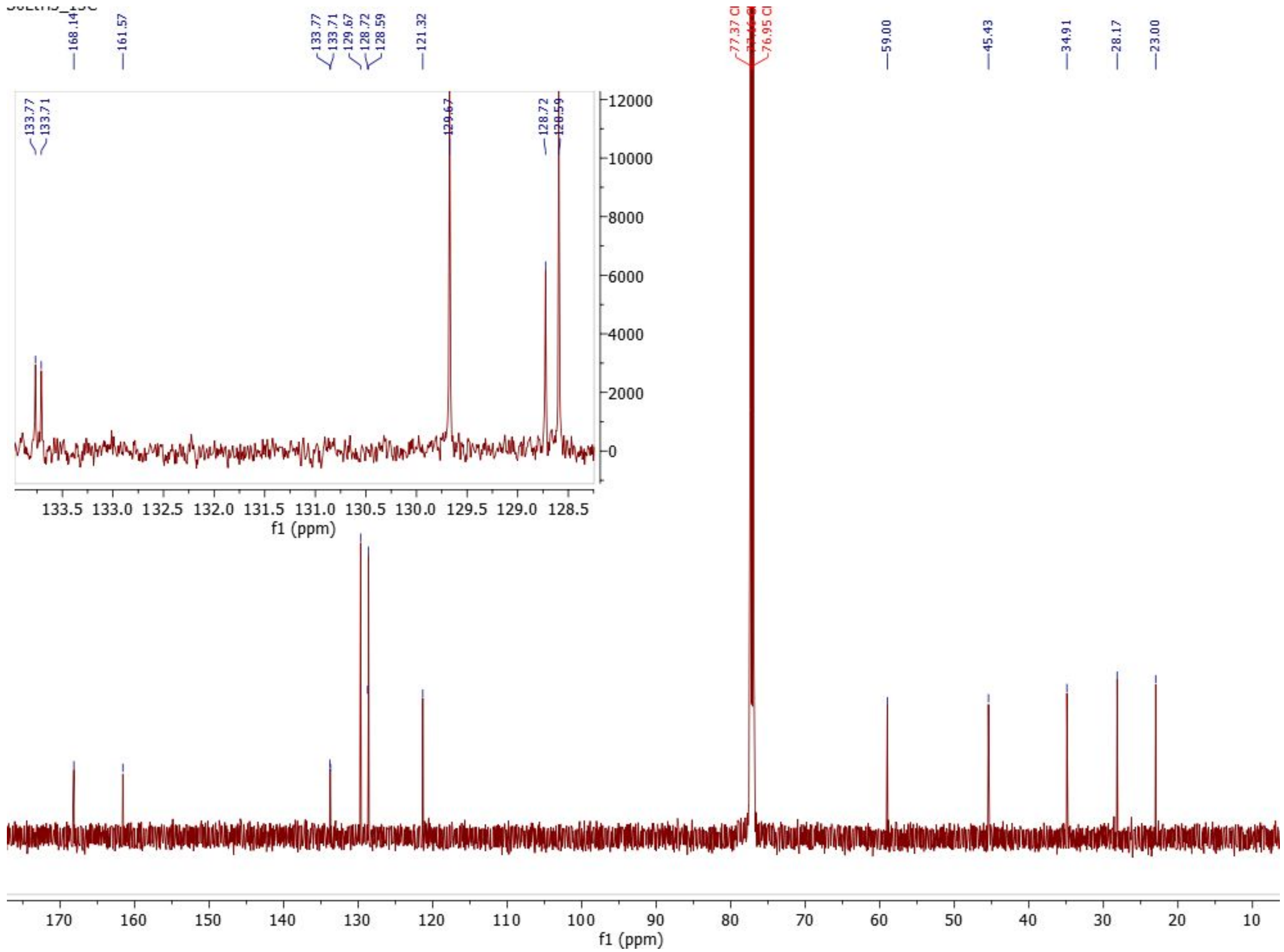

Figure S25. ^{13}C NMR Spectrum of **5** (150 MHz,

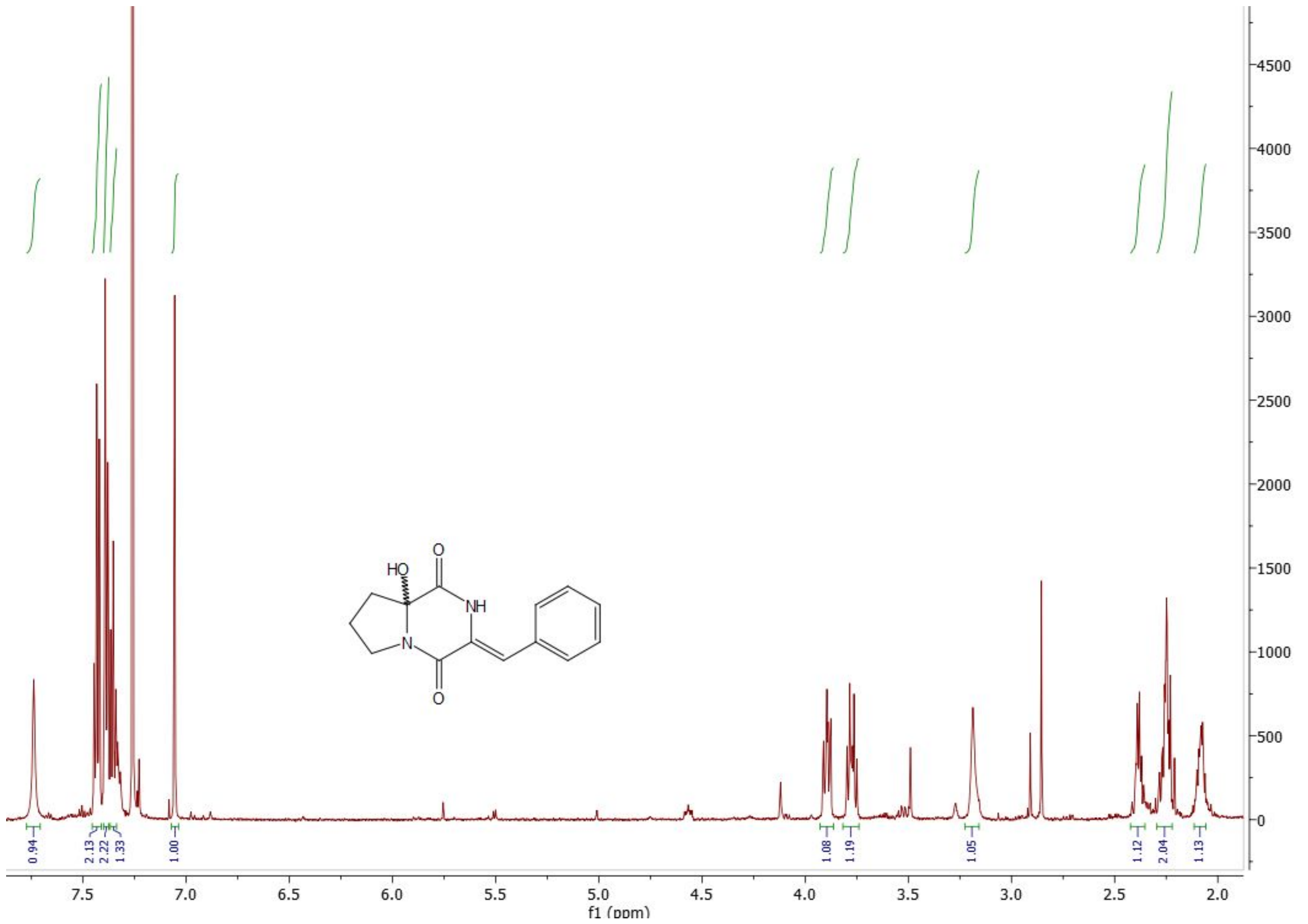

Figure S26. ^{1}H NMR Spectrum of **6** (600 MHz, $CDCl_3$)

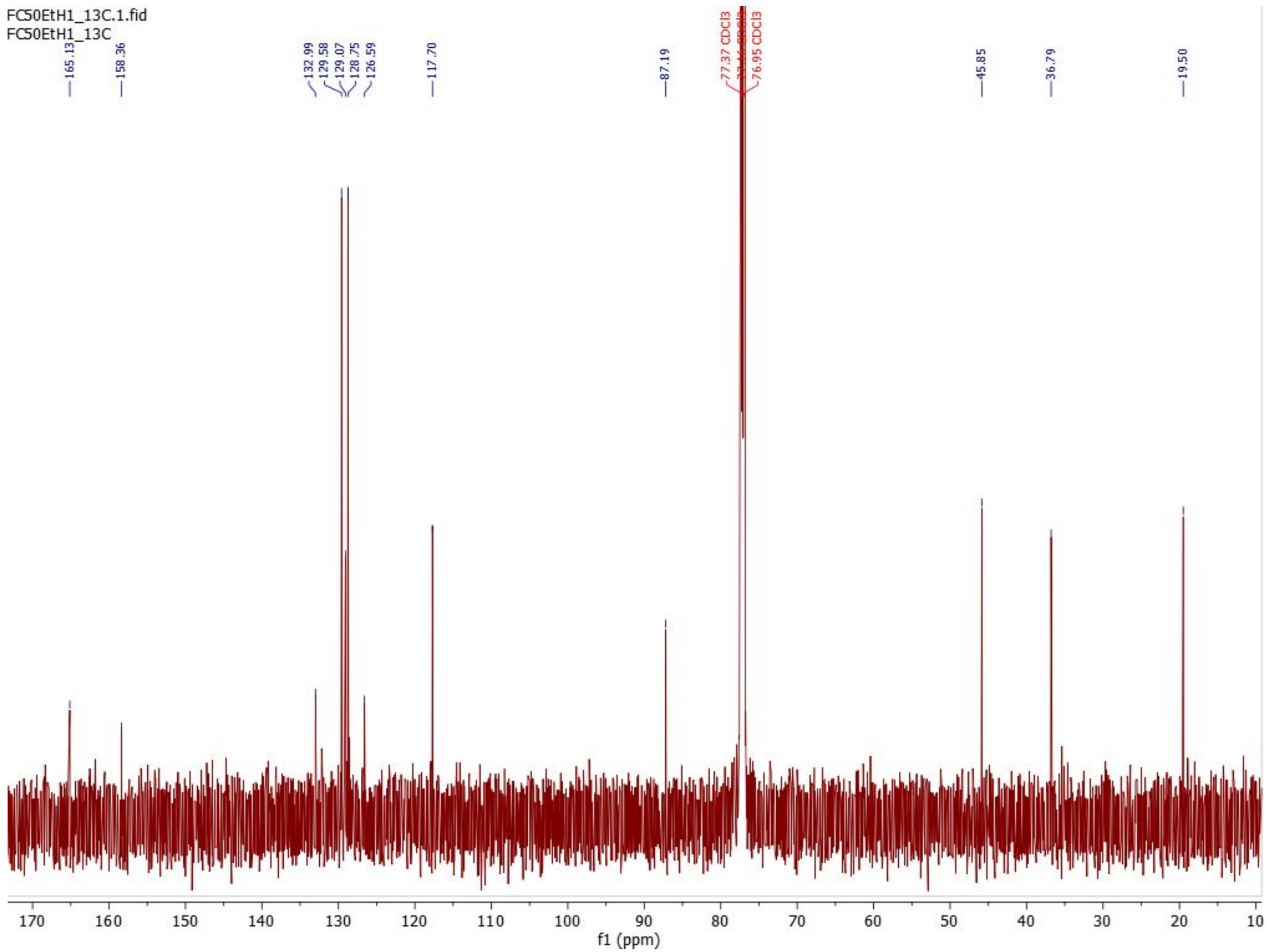

Figure S27. ^{13}C NMR Spectrum of **6** (150 MHz, CDC

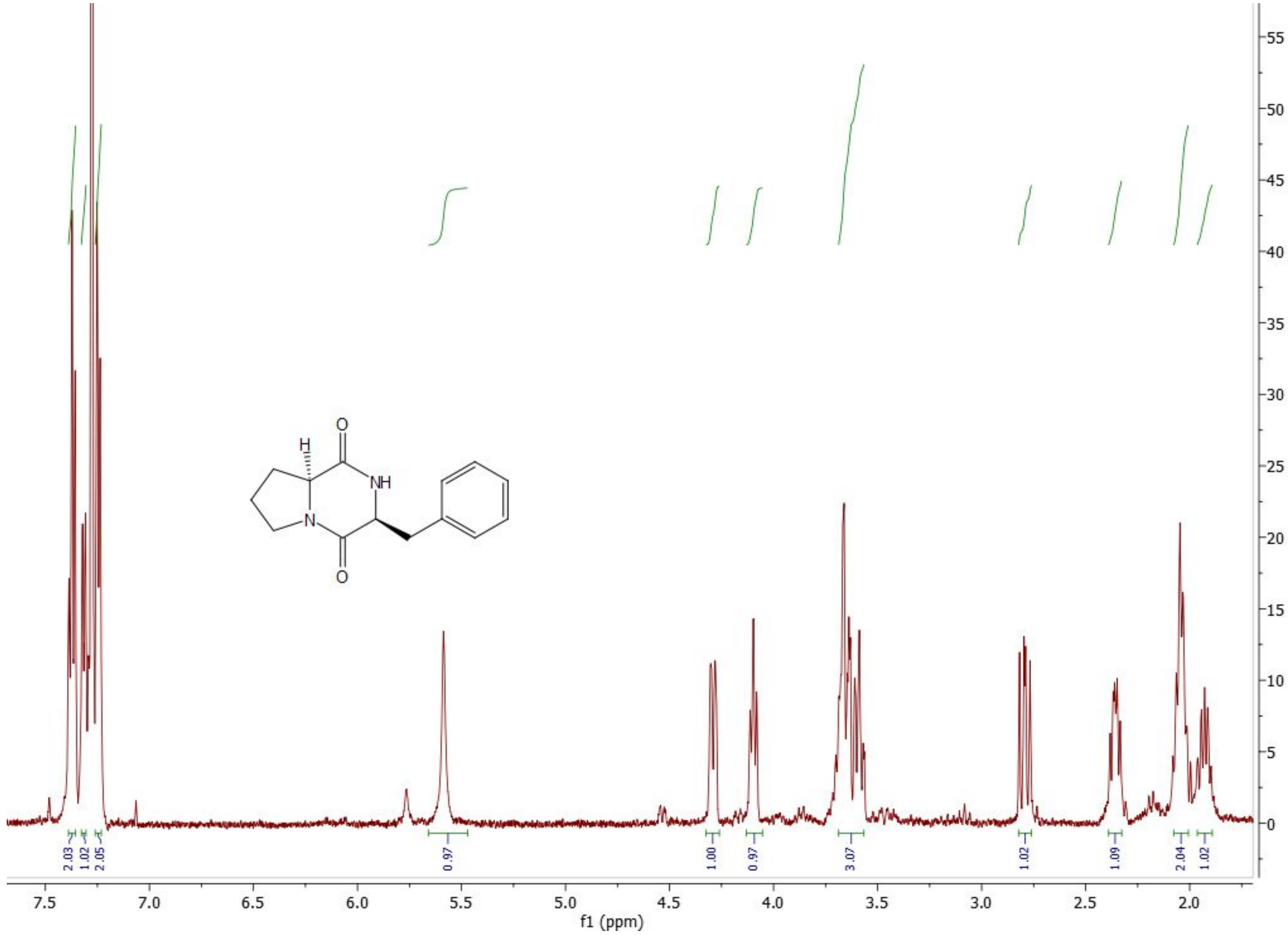

Figure S28. ^{1}H NMR Spectrum of **7** (500 MHz, $CDCl_3$)

Table S1. NMR spectroscopic data of broomeanamide B (**2**) in $CDCl_3$

Amino acid	position	$^{13}C^a$	^1H (mult., *J* in Hz)^b	HMBC
Pro	CO	173.5	-	-
	α-CH	55.8	4.04 (br t, 7.3)	-
	$β_1$-CH_2	28.8	1.07 (m)	-
	$β_2$-CH_2	28.8	0.55 (m)	-
	$γ_1$-CH_2	25.5	2.00 (m)	-
	$γ_2$-CH_2	25.5	1.52 (m)	-
	δ-CH_2	48.2	3.54 (t, 6.7)	28.8
N-MePhe	CO	-	-	-
	N-CH_3	28.8	2.83 (s)	173.5, 62.5
	α-CH	62.5	5.11 (dd, 10.3, 4.1)	-
	β-CH_2	34.0	3.02 (m)	129.6, 62.5
	γ-C (ar.)	138.3	-	-
	δ-CH_2 (ar.)	129.6	7.14 (d, 7.1)	129.6, 126.9, 34.0
	ε-CH_2 (ar.)	128.9	7.29 (t, 7.3)	138.3, 129.6
	ζ-CH (ar.)	126.9	7.22 (t, 7.6)	129.6
Ala	CO	174.9	-	-
	NH	-	8.85 (d, 7.5)	-
	α-CH	45.7	4.96 (m)	-
	β-CH_3	17.1	1.24 (d, 6.5)	174.9, 45.7
N-MeVal	CO	171.6	-	-
	N-CH_3	31.0	3.40 (s)	174.9, 56.8
	α-CH	56.8	5.31 (d, 10.6)	-
	β-CH	38.7	2.40 (m)	-
	$γ_1$-CH_3	19.8	1.00 (d, 6.5)	56.8, 38.7, 20.0
	$γ_2$-CH_3	20.0	0.95 (d, 6.5)	56.8, 38.7, 19.8
N-MeLeu^2	CO	-	-	-
	N-CH_3	29.2	2.87 (s)	171.6, 59.1
	α-CH	59.1	4.93 (m)	-
	$β_1$-CH_2	38.7	2.40 (m)	-
	$β_2$-CH_2	38.7	1.08 (m)	-
	γ-CH	25.5	1.45 (m)	-
	$δ_1$-CH_3	23.6	0.90 (d, 6.5)	38.7, 25.5, 22.7
	$δ_2$-CH_3	22.7	0.94 (d, 6.5)	38.7, 25.5
Val^1	CO	172.8	-	-
	NH	-	8.08 (d, 8.2)	-
	α-CH	54.6	4.90 (m)	-
	β-CH	33.6	1.78 (m)	-
	$γ_1$-CH_3	17.9	0.76 (d, 6.5)	54.6, 31.7, 20.5
	$γ_2$-CH_3	22.7	0.93(d, 6.5)	54.6, 31.7, 17.9
N-MeLeu^1	CO	-	-	-
	N-CH_3	30.9	2.92 (s)	172.8, 55.2
	α-CH	55.2	5.07 (brt, 7.5)	-
	$β_1$-CH_2	34.9	1.75 (m)	-
	$β_2$-CH_2	34.9	1.61	-
	γ-CH	25.5	1.45 (m)	-
	$δ_1$-CH_3	22.1	0.86 (d, 6.5)	34.9, 25.5, 20.1
	$δ_2$-CH_3	20.1	0.93 (d, 6.5)	34.9, 25.5, 22.1
Val^2	CO	170.9	-	-
	NH	-	8.14 (d, 8.1)	-
	α-CH	56.6	4.31 (br t, 8.5)	170.9
	β-CH	34.6	1.74 (m)	-
	$γ_1$-CH_3	18.9	0.82 (d, 6.5)	56.6, 34.6, 18.7
	$γ_2$-CH_3	18.7	0.85 (d, 6.5)	56.6, 34.6, 18.9

^{a}150 MHz;. ^{b}600 MHz.

Table S2. NMR spectroscopic data of broomeanamide C (**3**) in $CDCl_3$

Amino acid	position	$^{13}C^a$	^1H (mult., *J* in Hz)^b	HMBC
Pro	CO	173.3	-	-
	α-CH	55.2	4.06 (t, 6.7)	-
	$β_1$-CH_2	29.0	1.00 (m)	-
	$β_2$-CH_2	29.0	0.57 (m)	-
	$γ_1$-CH_2	25.3	1.97 (m)	-
	$γ_2$-CH_2	25.3	1.54 (m)	-
	δ-CH_2	48.1	3.53 (m)	-
N-MePhe	CO	-	-	-
	N-CH_3	29.0	2.82 (s)	173.3, 62.4
	α-CH	62.4	5.04(dd, 11.8, 3.0)	-
	β-CH_2	34.2	3.07 (m)	-
	γ-C (ar.)	138.2	-	-
	δ-CH_2 (ar.)	129.5	7.13 (d, 7.3)	126.8
	ε-CH_2 (ar.)	128.7	7.29 (t, 7.6)	129.5, 138.2
	ζ-CH (ar.)	126.8	7.21 (t, 6.7)	129.5
Ala	CO	-	-	-
	NH	-	8.80 (d, 8.3)	-
	α-CH	48.5	4.50 (m)	-
	β-CH_3	17.7	1.27 (d, 6.3)	172.9, 48.5
Val[2]	CO	172.0	-	-
	N-H	-	6.65 (d, 8.3)	-
	α-CH	54.7	4.66 (m)	-
	β-CH	33.8	2.10 (m)	-
	$γ_1$-CH_3	19.3	1.10 (d, 6.4)	54.7, 20.2
	$γ_2$-CH_3	20.2	0.97 (d, 6.4)	54.7, 19.3
N-MeLeu[2]	CO	-	-	-
	N-CH_3	29.4	2.85 (s)	172.0, 59.1
	α-CH	59.1	4.84 (dd, 11.0, 2.3)	-
	$β_1$-CH_2	38.3	2.41 (m)	-
	$β_2$-CH_2	38.3	1.30 (m)	-
	γ-CH	25.3	1.54 (m)	-
	$δ_1$-CH_3	23.3	0.89 (d, 6.4)	22.3
	$δ_2$-CH_3	22.3	0.94 (d, 6.4)	23.3
Ile	CO	172.4	-	-
	NH	-	8.45 (d, 8.3)	-
	α-CH	54.9	4.97 (m)	-
	β-CH	37.6	1.79 (m)	-
	γ-CH_3	22.8	0.92 (d, 6.3)	-
	γ-CH_2	28.4	1.32 (m)	-
	δ-CH_3	11.3	0.72 (t, 7.3)	37.6
N-MeLeu[1]	CO	-	-	-
	N-CH_3	30.4	2.92 (s)	172.4, 54.4
	α-CH	54.4	5.10 (t, 7.8)	-
	$β_1$-CH_2	34.7	1.74 (m)	-
	$β_2$-CH_2	34.7	1.62 (m)	-
	γ-CH	25.3	1.45 (m)	-
	$δ_1$-CH_3	22.3	0.86 (d, 6.4)	22.9
	$δ_2$-CH_3	22.8	0.92 (d, 6.4)	25.3
Val[1]	CO	-	-	-
	NH	-	7.97 (d, 8.3)	-
	α-CH	56.5	4.30 (t, 8.8)	-
	β-CH	34.7	1.74 (m)	-
	$γ_1$-CH_3	22.5	0.82 (d, 6.3)	56.5,34.7, 18.6
	$γ_2$-CH_3	18.6	0.85 (d, 6.3)	34.7, 22.5

^{a}150 MHz; ^{b}600 MHz.
